# Dysregulated ceramide metabolism in mouse progressive dermatitis resulting from constitutive activation of Jak1

**DOI:** 10.1016/j.jlr.2023.100329

**Published:** 2023-01-11

**Authors:** Yudai Iino, Tatsuro Naganuma, Makoto Arita

**Affiliations:** 1 Division of Physiological Chemistry and Metabolism, Graduate School of Pharmaceutical Sciences, Keio University, Tokyo, Japan; 2 Laboratory for Metabolomics, RIKEN Center for Integrative Medical Sciences, Yokohama, Japan; 3 Cellular and Molecular Epigenetics Laboratory, Graduate School of Medical Life Science, Yokohama City University, Yokohama, Japan

**Keywords:** Atopic dermatitis, ceramide desaturation, ceramides, LC-MS/MS, lipidomics, lipids, Cer[NDS], skin, sphingolipids, AD, atopic dermatitis, Cer, ceramides, Cer[EOS], ceramides containing ester-linked fatty acid, ω-hydroxy fatty acid, and sphingosine, Cer[NDS], ceramides containing nonhydroxy fatty acid and dihydrosphingosine, Cer[NS], ceramides containing nonhydroxy fatty acid and sphingosine, CerS, ceramide synthesis, *d*_3_, three deuterium atoms, *d*_7_, seven deuterium atoms, *d*_*9*_, nine deuterium atoms, DEGS, delta(4)-desaturase sphingolipid, DHS, dihydrosphingosine, ELOVL, elongation of long-chain fatty acids, JAK, Janus kinase, Kdsr, 3-ketoDHS reductase, LC, long-chain, LCB, long-chain base, LPC, lysophosphatidylcholine, MRM, multiple reaction monitoring, PNPLA1, patatin-like phospholipase domain-containing 1, SM, sphingomyelin, Sph, sphingosine, Spt, serine palmitoyl transferase, TEWL, transepidermal water loss, VLC, very long-chain, VLCFA, very long-chain fatty acid

## Abstract

Coordinated lipid metabolism contributes to maintaining skin homeostasis by regulating skin barrier formation, immune reactions, thermogenesis, and perception. Several reports have documented the changes in lipid composition in dermatitis, including in atopic dermatitis (AD); however, the specific mechanism by which these lipid profiles are altered during AD pathogenesis remains unknown. Here, we performed untargeted and targeted lipidomic analyses of an AD-like dermatitis model resulting from constitutive activation of Janus kinase 1 (Spade mice) to capture the comprehensive lipidome profile during dermatitis onset and progression. We successfully annotated over 700 skin lipids, including glycerophospholipids, ceramides, neutral lipids, and fatty acids, many of which were found to be present at significantly changed levels after dermatitis onset, as determined by the pruritus and erythema. Among them, we found the levels of ceramides composed of nonhydroxy fatty acid and dihydrosphingosine containing very long-chain (C22 or more) fatty acids were significantly downregulated before AD onset. Furthermore, in vitro enzyme assays using the skin of Spade mice demonstrated the enhancement of ceramide desaturation. Finally, we revealed topical application of ceramides composed of nonhydroxy fatty acid and dihydrosphingosine before AD onset effectively ameliorated the progression of AD symptoms in Spade mice. Our results suggest that the disruption in epidermal ceramide composition is caused by boosting ceramide desaturation in the initiation phase of AD, which regulates AD pathogenesis.

The skin is composed of the epidermis, dermis, and subcutaneous tissue. The epidermis provides a primary barrier to external stimuli and antigens. Defects in epidermal barrier function are risk factors for various cutaneous disorders, such as infectious diseases, ichthyosis, and atopic dermatitis (AD) (1, 2, 3). In the epidermis, keratinocytes constitute four layers (stratum basale, stratum spinosum, stratum granulosum, and stratum corneum) and play pivotal roles in maintaining the epidermal barrier. Keratinocytes proliferate in the stratum basale and migrate outwards by differentiating into layer-specific cell types. During differentiation, keratinocytes develop a barrier function by forming tight junctions and producing lamellar lipids. In the stratum corneum, lipid lamellae surround corneocytes, the terminally differentiated keratinocytes, to form a hydrophobic barrier. Thus, the proliferation and differentiation of keratinocytes should be appropriately regulated to maintain normal epidermal barrier function and skin homeostasis.

Various lipid molecules contribute to the regulation of keratinocytes and the maintenance of epidermal barrier function. Lipid lamellae in the stratum corneum function as hydrophobic barriers. The major components of lipid lamellae are cholesterol, FAs, and ceramides, among which ceramides are the most abundant (4). Ceramides are composed of FA and long-chain base (LCB) with structural variations in the number of double bonds, hydroxy groups, and carbon chain length and written by a combination of abbreviations corresponding to precursor structure (5, 6, 7) (supplemental Fig. S1). For example, the major ceramide classes in murine skin are composed of nonhydroxy FA and sphingosine or dihydrosphingosine written as Cer[NS] or Cer[NDS]. Lipid lamellae have a unique ceramide composition with a high proportion of very long-chain FA (VLCFA)-containing ceramides and acylceramides with an additional hydrophobic chain (linoleic acid) (8). Dysfunction of the enzymes responsible for producing lamellar ceramides causes severe barrier defects in humans and mice (9, 10, 11, 12, 13, 14, 15, 16, 17, 18, 19, 20). For example, mutations in the gene encoding patatin-like phospholipase domain-containing 1 (PNPLA1), which plays a pivotal role in the biosynthesis of acylceramide, cause autosomal recessive congenital ichthyosis (21). *Pnpla1* knockout mice show neonatal lethality due to epidermal permeability barrier defects (17, 21, 22). Although ceramides have received significant attention for a long time, other lipid species, such as triacylglycerol, ethanolamine plasmalogen, and LCB, also play important roles in maintaining barrier function and skin homeostasis (22, 23, 24, 25, 26). Thus, the skin lipid metabolic network elaborately regulates epidermal homeostasis as each lipid molecule exerts a characteristic bioactivity or physiological function.

AD is a chronic inflammatory dermatitis characterized by barrier disruption, pruritus, and excessive activation of type 2 immune response. Abnormalities in tight junctions and dysregulated keratinocyte differentiation are observed in AD (27, 28). In addition, diverse lipid metabolic changes are observed in AD lesional skin. For example, changes in the carbon chain length of lipids, such as ceramides, sphingomyelins (SMs), lysophosphatidylcholines (LPCs), or FAs, are observed in AD lesional skin, and the composition of ceramide classes is altered (29, 30, 31, 32). However, the specific mechanism by which the lipid profiles are altered during the pathogenesis of AD remains unknown.

In this study, we performed untargeted and targeted lipidomic analyses to determine the global lipid profiling in a progressive AD-like dermatitis of Spade mice that can spontaneously develop pruritic dermatitis by the mutation of the gene encoding Janus kinase 1 (Jak1), causing constitutive activation of Jak1 signaling (33). Notably, VLCFA-containing Cer[NDS] were selectively reduced with enhanced ceramide desaturation in the skin before the onset of AD symptoms, and topical application of Cer[NDS] effectively ameliorated the progression of AD symptoms in Spade mice.

## MATERIALS AND METHODS

### Experimental animals

Spade mice were kindly provided by H. Koseki (RIKEN Center for Integrative Medical Sciences, Laboratory for Developmental Genetics). Systemic *Jak1* mutation in Spade mice was produced by *N*-ethyl *N*-nitrosourea (ENU) mutagenesis protocol. The detailed method was described previously (33). Mice were maintained in specific pathogen-free environment with a 12-h light/dark cycle with water and a standard diet (CLEA Rodent Diet CE-2; CLEA Japan, Tokyo, Japan) provided ad libitum. All experiments involving the use of animals were approved by and performed in accordance with the Guidelines for Animal Experimentation of the Animal Use Committee at Keio University.

### Skin permeability assay

The skin permeability barrier was evaluated by measuring transepidermal water loss (TEWL) in the ears of mice using an evaporimeter (AS-VT100RS; Asahi Biomed, Yokohama, Japan). Measurements were taken three times for each ear, and the average value was calculated.

### Preparation of epidermis of murine ear

Murine ears were separated using forceps and incubated in RPMI containing 10% FBS, phosphatase inhibitor (Phosstop; Merck Millipore, Burlington, MA, USA), cOmplete protease inhibitor (Merck Millipore), and 1 mg/ml dispase (Thermo Fisher Scientific, Waltham, MA, USA) for 80 min at 37°C. After incubation, the epidermis was carefully separated from the dermis.

### Lipid extraction from murine ear

MULTI-BEADS SHOCKER MB1200 (YASUI KIKAI, Osaka, Japan) was used for pulverizing frozen ears. Crushed tissue was dissolved in 1 ml methanol and 500 μl chloroform containing the deuterium-labeled ceramide (1 μM *d*_9_-ceramides containing ester-linked FA, ω-hydroxy FA, and sphingosine (Cer[EOS]) d18:1/32:0/18:2; Cayman, Ann Arbor, MI, USA), followed by incubation at −30°C for 16 h. Samples were centrifuged (2,000 *g*, 4°C, 10 min), and 200 μl supernatants were dissolved in 100 μl methanol and 20 μl MilliQ water. After incubation (15 min, 20°C), the samples were centrifuged (2,000 *g*, 20°C, 10 min). The supernatants were applied for untargeted lipidomics.

The frozen epidermis and dermis were dissolved in 50 μl MilliQ water, 50 μl chloroform, and 100 μl methanol. Tissues were then dispersed using scissors. The total samples were mixed with 50 μl chloroform in an internal standard (300 nM *d*_7_-DHS d18:0, *d*_7_-Sph d18:1, *d*_3_-Cer[NS] d18:1/18:0, and *d*_3_-Cer[NDS] d18:0/18:0; Cayman). MilliQ water (50 μl) was added and vortexed. After centrifugation (10,000 *g*, 20°C, 1 min), the total volume of the precipitate was transferred to a glass insert (Agilent, Santa Clara, CA, USA) in a 2 ml vial. After drying, the dried samples were dissolved in 200 μl methanol. The samples (10 μl) were diluted with 190 μl of methanol and subjected to targeted lipidomics.

### LC-MS/MS analysis

An ACQUITY UPLC system (Waters, Milford, MA, USA) coupled with TripleTOF 6600 (Sciex, Framingham, MA, USA) was used for performing untargeted lipidomics. The conditions for LC separation and electrospray ionization were previously described (34). MS-DIAL was used for peak picking and alignment.

LCMS8060 (Shimadzu, Osaka, Japan) was used for targeted ceramide and SM analysis. The condition of lipidomics was previously described by Ohno *et al.* (22). A reverse-phase column (ACQUITY UPLC HSS T3 1.8 μm, LC column 50 × 2.1 mm) was used. Gradient elution of mobile phase A (acetonitrile/MilliQ water [3:2, v/v] containing 10 mM ammonium acetate) and mobile phase B (acetonitrile/isopropanol [1:9, v/v] containing 10 mM ammonium acetate) were used for LC separation. The gradient was as follows: 0–18 min, linear gradient from 40% B to 100% B; 18–23 min, 100% B; 23–25 min, 40% B. The column temperature was 55°C, the flow rate was 0.30 ml/min, and the injection volume was 1 μl. The multiple reaction monitoring (MRM) mode was applied to detect ceramides and SMs. MRM transitions and collision energies are listed in supplemental Table S1. For quantification, Cer[NS] d18:1/16:0, Cer[NS] d18:1/24:0, Cer[NDS] d18:0/16:0, Cer[NDS] d18:0/24:0, SM d18:1/16:0, and SM d18:1/24:0 (Avanti) were used for 8-point calibration curve method.

LCMS8060 (Shimadzu) was used for targeted LCB analysis. A reverse-phase column [Kinetex 2.6 μm C8 100 Å, LC Column 150 × 2.1 mm] was used for LC separation with a gradient elution of mobile phase A (water 0.2% formate) and mobile phase B (acetonitrile). The gradient was as follows: 0–1 min, 10% B; 1–4 min, linear gradient to 40% B; 4–10 min, 75% B; 10–12 min, linear gradient to 100% B; 12–27 min, 100% B; 27–30 min, 10% B. The flow rate was 0.40 ml/min, the column temperature was 40°C, and the injection volume was 1 μl. The MRM mode was applied to detect LCBs. MRM transitions and collision energies are listed in supplemental Table S2. For quantification, DHS d18:0 and Sph d18:1 (Avanti) were used for the 8-point calibration curve method.

### In vitro measurement of enzyme activity

Frozen ears were pulverized with a metal cone using a MULTI-BEADS SHOCKER MB1200 and dissolved in 500 μl HEPES buffer (50 mM HEPES/NaOH [pH 7.5], 150 mM NaCl, 10% glycerol) containing cOmplete protease inhibitor (Merck Millipore), 1 mM dithiothreitol, and 1 mM Phosstop. The samples were sonicated for 10 s at three times. After centrifugation (200 *g*, 4°C, 3 min), the supernatants were collected in new tubes. Proteins were quantified by BCA analysis (Thermo Fisher Scientific). The samples were then subjected to in vitro protein activities. For the examination of de novo sphingolipid metabolic activity (serine palmitoyl transferase (Spt) and 3-ketoDHS reductase (Kdsr)), 200–300 μg of proteins were incubated with 4 mM ^13^C_3_, ^15^N-_L_-serine (Merck Millipore), and 20 μM C16:0-CoA (Merck Millipore) for 1 h at 37°C. The assay mixture, with a total volume of 40 μl, also contained 1 mM NADPH (Merck Millipore), 500 μM pyridoxal 5′-phosphate (Merck Millipore), cOmplete protease inhibitor, Phosstop, 50 mM HEPES/NaOH (pH 7.5), 150 mM NaCl, and 10% glycerol. To examine CerS and desaturation activity, 200–300 μg proteins were incubated with 5 μM *d*_7_-Sph d18:1 or *d*_7_-DHS d18:0 and 20 μM C16:0-CoA or C24:0-CoA (Merck Millipore) for 1 h at 37°C. The assay mixture, with a total volume of 40 μl, also contained cOmplete protease inhibitor, Phosstop, 50 mM HEPES/NaOH (pH 7.5), 150 mM NaCl, and 10% glycerol. All in vitro sphingolipid biosynthesis reactions were stopped by the addition of 50 μl chloroform and 100 μl methanol. After vortexing, the total sample was mixed with 50 μl chloroform containing an internal standard (30 nM *d*_7_-DHS d18:0, *d*_7_-Sph d18:1, *d*_3_-Cer[NS] d18:1/d18:0, and *d*_3_-Cer[NDS] d18:0/18:0). MilliQ water (60 μl) was added, and samples were vortexed. After centrifugation (10,000 *g*, 20°C, 1 min), the total volume of the precipitate was transferred to a glass insert (Agilent) in a 2 ml vial. After drying, the dried samples were dissolved in 50 μl methanol and subjected to targeted lipidomics.

### Quantitative RT-PCR

The frozen tissue was pulverized with a metal cone using a MULTI-BEADS SHOCKER MB1200. RNeasy Mini Kit (Qiagen, Venlo, Netherlands) was used for total RNA preparation. 500 ng of RNA was subjected to reverse transcription using ReverTra Ace qPCR RT Master Mix (TOYOBO, Osaka, Japan). THUNDERBIRD NEXT SYBR qPCR MIX (TOYOBO) was used for quantitative RT-PCR. The forward (F) and reverse (R) primer pairs used in this study are listed in supplemental Table S3.

### Immunoblotting

The frozen tissue was pulverized with a metal cone using MULTI-BEADS SHOCKER MB1200 and dissolved in 1% SDS buffer (62.5 mM Tris/HCl [pH 6.8], 1% SDS, 10% glycerol, 5% 2-mercaptoethanol, 0.02% bromophenol blue, cOmplete protease inhibitor, and Phosstop). The samples were sonicated for 10 s at three times. After centrifugation (10,000 *g*, 20°C, 3 min), supernatants were collected in new tubes. Proteins were quantified by BCA analysis (Thermo Fisher Scientific). 10–20 μg proteins were applied to SDS-PAGE and subsequently electrotransferred onto PVDF membranes (Merck Millipore). Immunoblotting was performed using primary and secondary antibodies: rabbit anti-Actin (A2066; 1:3000; Merck Millipore), rabbit anti-delta 4-desaturase, sphingolipid 1 (DEGS1; PA5-42741; 1:1000 dilution; Thermo Fisher Scientific), and HRP-conjugated anti-rabbit IgG F(ab’)_2_ fragment (ab6721, 1:5000 dilution; Abcam, Cambridge, UK). ECL Western blotting substrate (Bio-Rad, Berkeley, CA, USA) was used for labeling.

### Topical application of ceramide

Cer[NDS] d18:0/24:0 (1 μg; Avanti, Alabaster, AL, USA) or Cer[NDS] d18:0/16:0 (1 μg; Avanti) in acetone and olive oil (4:1, v/v) 20 μl or solvent alone as control were applied every other day to the ears from 4 to 16 weeks of age. The clinical severity score was determined as the average of the individual scores for symptoms in the right and left ears (33, 35). Symptoms were graded as 0 (none); 1 (mild; erythema, hemorrhage, and excoriation); 2 (moderate; erosion, crust, and rhagades on the ear); or 3 (severe; thickening/lichenification and deformed/defective ears). Disease onset was defined as a clinical score > 0.5.

### Histological and immunohistological analysis

Murine ear skin was fixed with 4% paraformaldehyde at 4°C overnight, embedded in paraffin, and sectioned at 5–10 μm for H&E staining. For immunohistochemistry, fixed ear specimens were sectioned at 5 μm, and antigens were retrieved with 10 mM sodium citrate buffer (pH 6.0) by autoclaving for 15 min at 120°C. After blocking with 1% BSA in PBS for 1 h at 20°C, the slides were stained with Krt10 (ab76318; Abcam), Ki67 (ab15580; Abcam), Lor (ab85679; Abcam) or Krt5 (905504; Biolegend), followed by incubation with Alexa Fluor 488 goat anti-rabbit IgG (H+L) (A-11006; Thermo Fisher Scientific) secondary antibodies. The slides were counterstained with DAPI (ab104139; Abcam).

The transmission electron microscopic analysis of lipid lamellae using ruthenium tetroxide was conducted as described previously (21, 36). For transmission electron microscopy, mouse ear skin was fixed with 2% paraformaldehyde and 2% glutaraldehyde in 0.1 M cacodylate buffer at 4°C. Then, samples were fixed in 2% osmium tetroxide solution at 4°C for 2 h and subsequently postfixed in 2% ruthenium tetroxide solution and 0.25% potassium hexacyanoferrate at 4°C for 2 h under pressure. After dehydration in graded ethanol solutions (30–100%), samples were infiltrated with propylene oxide three times for 15 min. Then, samples were embedded in the epoxy resin (EPON812; EM Japan, Tokyo, Japan). After making ultrathin ear sections (80–90 nm) by 2088 ULTRATOME Ⅴ (LKB, Vienna, Austria), samples were stained with 2% uranyl acetate for 15 min and lead stain solution (Merck Millipore) for 2 min. Transmission electron microscopy observation was conducted by H-7600 (HITACHI, Tokyo, Japan). Images were taken at 100 kV of acceleration voltage.

### Analytical validation

Results are expressed as the mean + standard error. Data were analyzed using Excel and R statistical software. They were statistically analyzed using Student’s *t-*test, Dunnett's test, and Mann-Whitney’s *U* test. *P* < 0.05 was considered to be statistically significant.

## RESULTS

### Global lipid profiling during AD pathogenesis in Spade mice

**Fig. 1:**
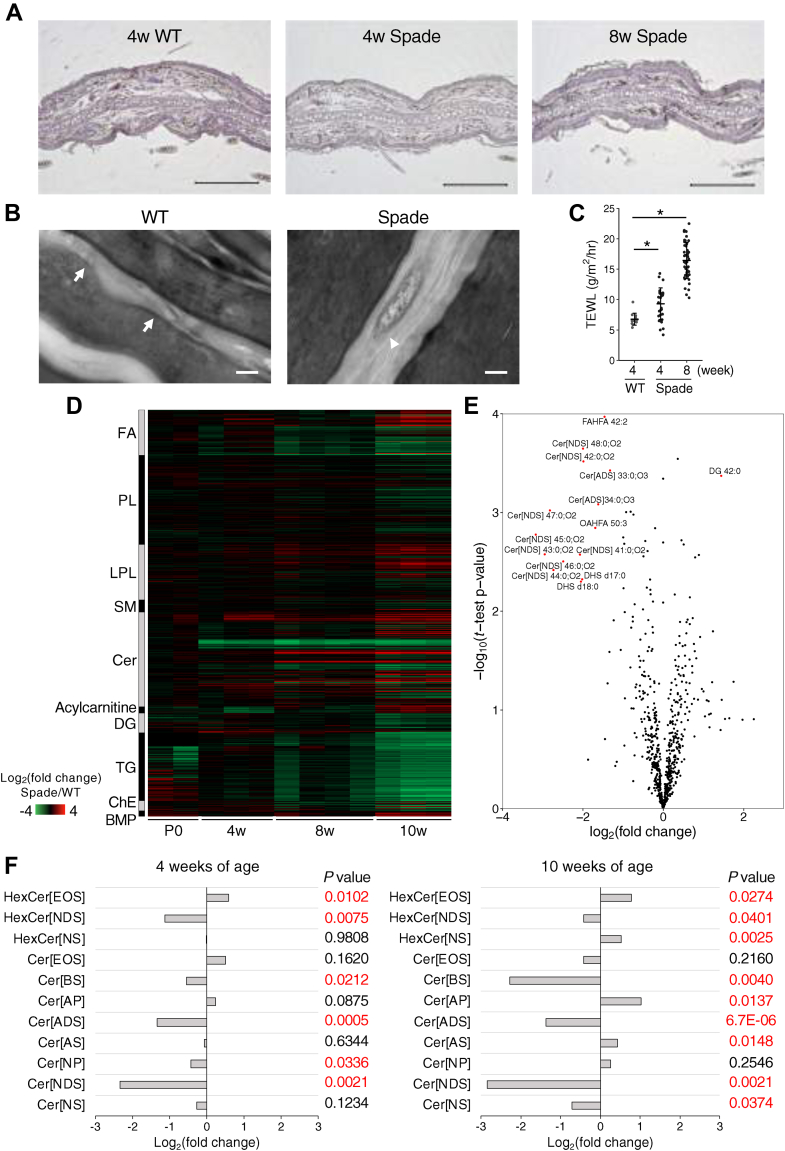
Time-course profiling of the lipid metabolic changes during dermatitis development. A: Ear sections of WT or Spade mice stained with H&E. Scale bars: 200 μm. B: Ear photographs of WT or Spade mice at 4 weeks of age by transmission electron microscopy. Normal lamellar structure (arrow) and some aggregates like remaining substances of lamellar bodies (arrowheads) were observed. Scale bars: 100 nm. C: Analysis of transepidermal water loss (TEWL) in WT and Spade mice at 4 or 8 weeks of age (n = 14–48) via Dunnett’s test, ^∗^*P* < 0.05, mean ± standard error (SE). D: Heatmap of lipid signal intensity. Each molecular species was semi-quantified via untargeted lipidomics and normalized by tissue weight. Color is proportional to the intensity of the change in metabolites. Each individual data point was expressed relative to an average of WT. P0 (n = 2); 4, 10 weeks (n = 3); 8 weeks (n = 4). E: Volcano plot showing the changes in the relative percentage of annotated lipids in Spade mice versus WT mice at 4 weeks of age. F: Fold changes in ceramide classes in Spade mice compared with those in age-matched WT.

Hyperactivation of Jak1 caused by the single amino acid substitution in Spade mice leads to Th2 dermatitis (33). Progressive dermatitis develops as desquamation and redness of the ears at approximately 8 weeks of age. At 10 weeks of age, serum IgE and IgG1 levels are increased, and Th2 cytokines, such as IL-4, IL-5, and IL-13, produced by CD4^+^ cells are upregulated, followed by elevated serum histamine levels at 12 weeks of age. Skin lesions manifested as epidermal hyperplasia at 8 weeks, while there were few morphological changes at 4 weeks of age (Fig. 1A). Transmission electron microscopic analysis showed that in Spade mice at 4 weeks of age, normal lipid lamellar structures were observed in most parts of the sections, as shown in WT. However, some aggregates, like remaining substances of lamellar bodies, were found in the Spade lipid lamellae occasionally (Fig. 1B). TEWL, the barrier function readout, was significantly elevated in Spade mice at 4 weeks of age (Fig. 1C), suggesting that barrier defects had occurred before disease onset. To analyze the comprehensive lipid profile at each stage of pathogenesis, WT and Spade skin tissues at P0, 4, 8, and 10 weeks of age were applied to untargeted lipidomics, which resulted in monitoring 745 lipid species annotated by negative and positive ion modes (supplemental Fig. S2A). Principal component analysis revealed that the lipidome profile of Spade mice differed from that of WT mice, especially at 8 and 10 weeks of age (supplemental Fig. S2B). In the epidermis of patients with AD, the proportion of very long-chain (VLC) Cer[NS] (44:1;O2–50:1;O2), SMs (44:1;O2–46:1;O2), and LPCs (24:0–30:0) decreases with a simultaneous increase in long-chain (LC) Cer[NS] (32:1;O2–40:1;O2), SM (32:1;O2), and LPCs (14:0–22:0) (32). In Spade mice at 10 weeks of age, reduced levels of VLC Cer[NS] (42:1;O2–50:1;O2) and LPCs (24:0–26:0) were evident (supplemental Fig. S2C, D). In addition, Cer[NS] (32:1;O2–40:1;O2) and LPCs (16:0, 18:0) increased. In contrast, the levels of SMs (32:1;O2, 36:1;O2, and 38:1;O2) decreased, while VLC SM (44:1;O2) increased, which was different from the lipid profile in the human AD region (supplemental Fig. S2E). Furthermore, increased unsaturated FAs and reduced saturated VLCFAs (22:0–28:0), shown in the human AD region, were confirmed in Spade mice at 10 weeks of age (29, 30) (supplemental Fig. S2F, G). Berdyshev *et al.* showed that in the epidermis of patients with AD, mRNA expression levels of FA elongases, elongation of very long-chain FA (*ELOVL3/6*), were decreased, which contributed to proportional changes in carbon chain length in AD skin lipids (32). At 10 weeks of age, the mRNA expression of *Elovl6* was reduced significantly and *Elovl3/4/5* levels tended to decrease compared to WT (supplemental Fig. S2H). Interestingly, in Spade mice at 4 weeks of age, we found a cluster with a large reduction in ceramide species, although there was no obvious change in other lipid classes (Fig. 1D). Ceramides are classified into various classes according to the different combinations of their precursors (LCB and FA) (5, 6, 7) (supplemental Fig. S1). The volcano plot showed a dramatic and significant decrease in the Cer[NDS] species in Spade mice at 4 weeks of age, before the onset of AD (Fig. 1E). Ishikawa *et al.* showed the downregulation of ceramide species such as acylceramide {Cer[EOS], ceramides containing ester-linked FA, ω-hydroxy FA, and phytosphingosine (Cer[EOP]), ceramides containing ester-linked FA, ω-hydroxy FA, and 6-hydroxysphingosine (Cer[EOH])}, ceramides containing nonhydroxy FA and phytosphingosine (Cer[NP]), and ceramides containing nonhydroxy FA and 6-hydroxysphingosine (Cer[NH]) with the upregulation of ceramides containing α-hydroxy FA and sphingosine (Cer[AS]) in addition to Cer[NDS] reduction in the affected sites of patients with AD (31). Among them, only Cer[NDS] decreased in Spade mice at 4 weeks of age with HexCer[NDS], one of their precursors, although the reduction of Cer[EOS] and upregulation of Cer[AS] were observed in Spade mice at 10 weeks of age (Figs. 1F, S2I and supplemental Table S4).

### VLCFA-containing Cer[NDS] were selectively reduced in the Spade epidermis before AD onset

**Fig. 2:**
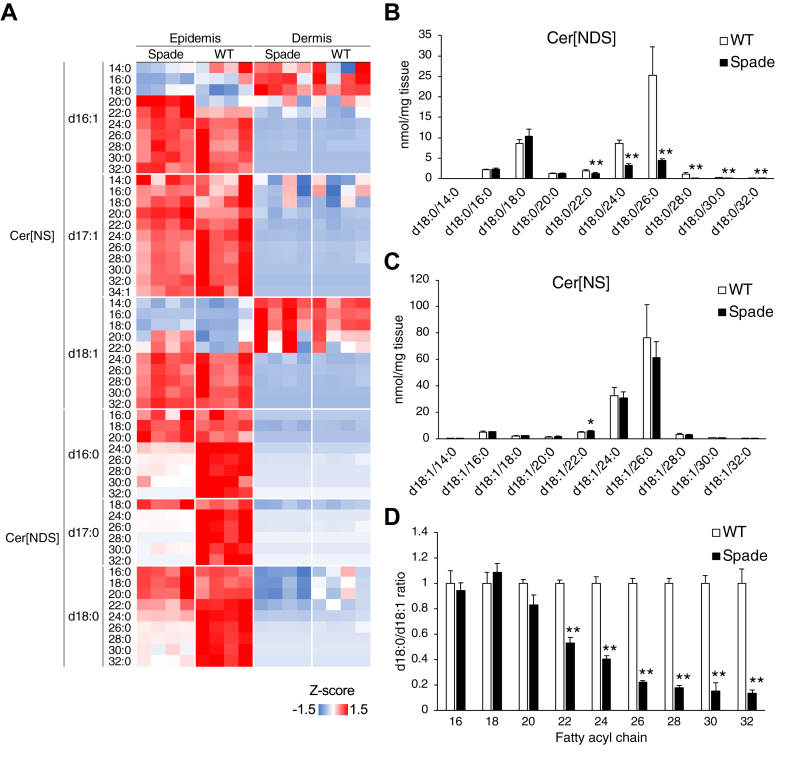
Quantification of Cer[NS] and Cer[NDS] in epidermis or dermis. A: Z-score of ceramide quantification results. Epidermis is separated from dermis in 4-week-old WT and Spade mice. B, C: Levels of Cer[NDS] and Cer[NS] in epidermis were expressed as concentration per ear weight. D: Ratios of Cer[NDS] to Cer[NS] were expressed as each fatty acyl carbon chain length of ceramide. n = 4, mean + SE, student’s *t-*test, ^∗^*P* < 0.05, ^∗∗^*P* < 0.01. Cer[NDS], ceramides containing nonhydroxy FA and dihydrosphingosine; Cer[NS], ceramides containing nonhydroxy FA and sphingosine.

Ceramides have a crucial role in regulating skin barrier function, and it has been shown that the disruption of ceramide metabolism leads to skin inflammation (37, 38). Since ceramide metabolism was altered in Spade mice before AD onset, we focused on its metabolism. To ascertain whether the disruption of ceramide metabolism occurred in the epidermal or dermal tissue of the ear, we performed MRM-based targeted analysis for the quantification of Cer[NS] and Cer[NDS] containing C16–18 LCBs and C14–32 FAs in the epidermis and dermis prepared from WT and Spade mice at 4 weeks of age. In Cer[NS] with d16:1 or d18:1 LCBs, ceramides with C24 or more FAs dominantly exited the epidermis, whereas those with C18 or fewer FAs were dominant in the dermis (Fig. 2A). Cer[NS] with d17:1 LCB and Cer[NDS] were abundant in the epidermis. Among them, the levels of Cer[NDS] containing VLCFAs (C22 or more) were dramatically decreased in the Spade epidermis, although there was no difference in the levels of Cer[NS] between WT and Spade mice in both the epidermis and dermis. In Cer[NDS], d18:0/18:0, d18:0/24:0, and d18:0/26:0 were mainly present in the epidermis and the amounts of d18:0/24:0 and d18:0/26:0 were significantly decreased (d18:0/24:0, 39%; d18:0/26:0, 17% vs. WT) although d18:0/18:0 was not reduced (d18:0/18:0, 119% vs. WT) (Fig. 2B). None of the Cer[NS] containing d18:1 LCB declined in the Spade epidermis although Cer[NS] d18:1/22:0 was slightly upregulated (d18:1/22:0, 116% vs. WT) (Fig. 2C). Selective reduction of Cer[NDS] caused a significant decrease in the Cer[NDS]/Cer[NS] ratio in ceramides containing VLCFAs in the Spade epidermis (Fig. 2D). In addition, the amount of dihydrosphingosine (DHS), a precursor of Cer[NDS], declined significantly (d16:0, 13%; d17:0, 8.8%; d18:0, 17% vs. WT) in the Spade epidermis compared with WT, and the amount of sphingosine (Sph) also declined (d16:1, 62%; d17:1, 46%; d18:1, 64% vs. WT) (supplemental Fig. S3A). The levels of DHS (d18:0) and Sph (d16:1, d17:1, and d18:1) in the dermis did not change (supplemental Fig. S3B). In the Spade epidermis, the total amount of Cer[NDS] declined significantly, although that of Cer[NS] did not change (supplemental Fig. S3C, D). Furthermore, the total amount of SM containing Sph d18:1, the end product of Cer[NS], was significantly upregulated (139% vs. WT) (supplemental Fig. S3E). Altogether, VLCFA-containing Cer[NDS] were selectively reduced in the epidermis, suggesting that aberrant sphingolipid metabolism might impair skin homeostasis in Spade mice before the onset of AD.

### Degs1-associated aberrant ceramide metabolism in Spade mice

**Fig. 3:**
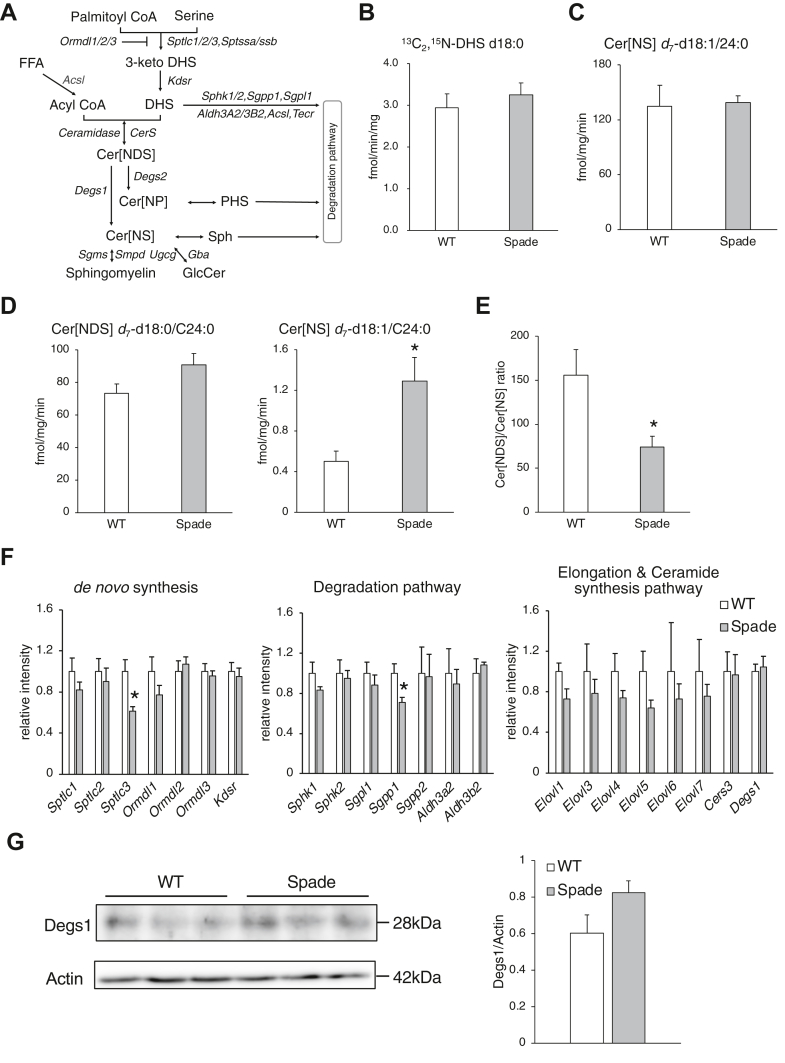
Analysis of sphingolipid metabolic protein activity. A: Ceramide metabolic pathway. Lipids (black) and genes (grey) were described. B: ^13^C_3_,^15^N-serine (4 mM) was incubated for 60 min at 37°C with 20 μM C16:0-CoA in the homogenates of murine ear. NADPH (1 mM) and pyridoxal 5′-phosphate (PLP; 500 μM) were used as cofactors. ^13^C_2_,^15^N-d18:0 was detected via LC-MS/MS. n = 3. C, D: *d*_7_-Sph d18:1 or *d*_7_-DHS d18:0 was incubated with C24:0-CoA. The products were quantified via LC-MS/MS. NADH (1 mM) was used as a cofactor. n = 3. D: Cer[NS] *d*_7_-d18:1/24:0 was also measured as an indicator of ceramide desaturation via incubation with *d*_7_-DHS d18:0 and C24:0-CoA. E: Product ratio of (*d*_7_-d18:0/24:0)/(*d*_7_-d18:1/24:0) via incubation with *d*_7_-DHS d18:0 and 24:0-CoA in ear homogenates. n = 3. F: mRNA expression levels in the epidermis of 4-week-old WT and Spade mice. Sphingolipid metabolic genes in the epidermis were analyzed via qPCR. Levels of gene expressions were normalized by levels of *Gapdh*. Data were expressed relative to the average level of each gene in WT epidermis. n = 4. G: Western blotting analysis of ear lysates revealed Delta 4-desaturase, sphingolipid (Degs1) and Actin expression levels. Bands were quantified using the Image-J software. mean + SE, student’s *t-*test, ^∗^*P* < 0.05, ^∗∗^*P* < 0.01. Cer[NS], ceramides containing nonhydroxy FA and sphingosine; DHS, dihydrosphingosine; Sph, sphingosine; qPCR, quantitative PCR.

We next performed in vitro measurements of the enzyme activities to investigate the mechanism by which Cer[NDS] were selectively decreased in Spade mice. In the sphingolipid metabolic pathway, acyl-CoA is condensed with serine to generate 3-ketoDHS by serine palmitoyl transferase (SPT), which is reduced to DHS by 3-ketoDHS reductase (KDSR) (39) (Fig. 3A). DHS is acylated to Cer[NDS] via ceramide synthase (CERS). Subsequently, Cer[NDS] are reduced to Cer[NS] by DEGS. We investigated whether the reduction of Cer[NDS] in Spade mice is attributed to either decreased de novo synthesis (Spt, Kdsr), decreased ceramide synthesis (CerS), or increased ceramide desaturation (Degs). We measured the de novo synthesis activity using C16:0-CoA and ^13^C_3_,^15^N-serine as substrates. As a result, the production of DHS (^13^C_2_,^15^N-d18:0) was not changed in the Spade mice tissue at 4 weeks of age compared to WT (Fig. 3B). This result showed that *de nov*o synthesis activity did not decrease in 4-week-old Spade mice compared to WT. CerS activity was measured using C24:0-CoA, which is recognized by CerS2/3 as a substrate (40, 41). When *d*_7_-Sph (*d*_7_-d18:1) was incubated with C24:0-CoA, there was no difference in the production of Cer[NS] *d*_7_-d18:1/24:0 between the WT and Spade mice tissue homogenates (Fig. 3C). Similarly, the production of Cer[NDS] *d*_7_-d18:0/24:0 did not change when *d*_7_-DHS (*d*_7_-d18:0) was used, suggesting that the CerS activity was not reduced in Spade mice (Fig. 3D). In contrast, the amount of Cer[NS] *d*_7_-d18:1/24:0, which is produced by desaturation of Cer[NDS] *d*_7_-d18:0/24:0, increased approximately 2-fold in Spade mice, resulting in a reduced (Cer[NDS] *d*_7_-d18:0/24:0)/(Cer[NS] *d*_7_-d18:1/24:0) ratio (Fig. 3D, E). We also used C16:0-CoA, which is recognized by CerS5/6 for the measurement of CerS and desaturation, and found normal CerS and enhanced ceramide desaturation (42, 43) (supplemental Fig. S4A–C). These results indicate the upregulation of ceramide desaturation and could explain why the amount of Cer[NDS] was reduced in the Spade skin before AD onset. To unveil the mechanism of ceramide desaturation enhancement in Spade mice, we investigated the expression of epidermal genes related to ceramide metabolism. The mRNA levels of *Degs1*, involved in ceramide desaturation, were not significantly upregulated, although some sphingolipid metabolic genes were slightly downregulated (*Sptlc3*, 61%; *Sgpp1*, 71%) in the Spade epidermis at 4 weeks of age (Fig. 3F). In addition, there was no significant change in Degs1 protein expression in Spade mice at 4 weeks of age, whereas ceramide desaturation was enhanced approximately 2-fold in Spade mice compared with WT (Figs. 2D and 3G).

### Topical application of Cer[NDS] delays dermatitis onset and development

**Fig. 4:**
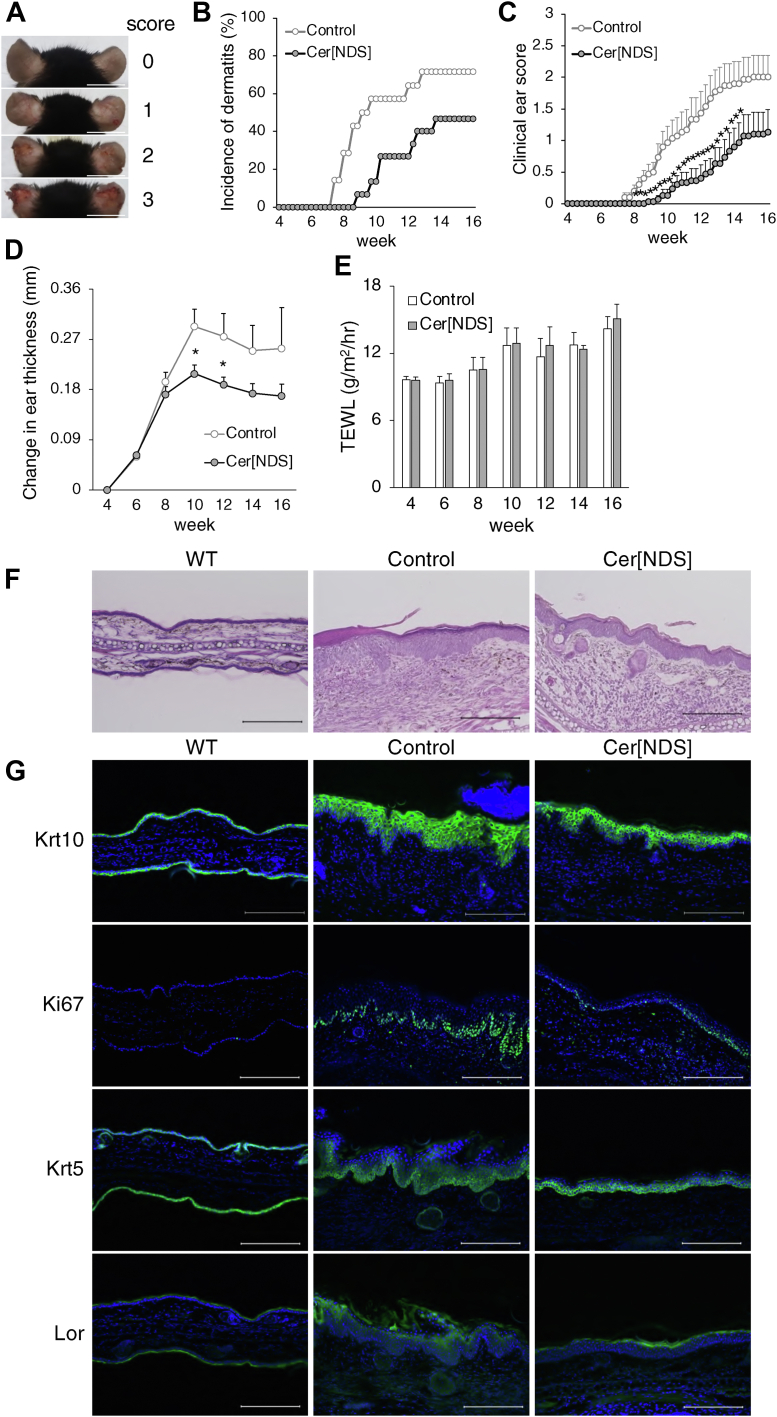
Analysis of Spade mice phenotype via the topical application of Cer[NDS] d18:0/24:0. A: Dermatitis in Spade mice was scored by acanthosis and erythema. Scale bars: 1 cm. B, C: Analyses of dermatitis incidence and clinical scores of Spade mice treated with Cer[NDS] d18:0/24:0 in acetone : olive oil (4:1, v/v) solution or solvent alone as control. Mann-Whitney’s *U* test. D, E: Analyses of change in ear thickness and TEWL in mice treated with Cer[NDS] d18:0/24:0 or solvent alone. Student’s *t-*test. F: Ear sections of WT mice, control or Cer[NDS] d18:0/24:0-treated Spade mice stained with H&E. Scale bars: 200 μm. G: Ear sections of WT mice, control or Cer[NDS] d18:0/24:0-treated Spade mice 8 weeks after treatment were stained with anti-Krt10, anti-Ki67, anti-Krt5, anti-Lor (green), and DAPI (blue). Scale bars: 200 μm. n = 15, mean + SE, ^∗^*P* < 0.05. Cer[NDS], ceramides containing nonhydroxy FA and dihydrosphingosine; TEWL, transepidermal water loss.

To investigate whether Cer[NDS] containing VLCFAs suppress AD pathogenesis, Cer[NDS] d18:0/24:0 in acetone: olive oil (4:1, v/v) 20 μl or solvent alone were topically applied to the skin of Spade mice every alternate day from 4 weeks of age. The onset and progression of dermatitis were delayed by the treatment with Cer[NDS] d18:0/24:0, and by 16 weeks of age, only 50% of Cer[NDS] d18:0/24:0 treated mice had developed the disease, although 70% of nontreated mice had developed it (Fig. 4A, B). The clinical score evaluated by acanthosis and erythema in the ear also improved with Cer[NDS] d18:0/24:0 treatment (Fig. 4C). Consistent with these findings, ear and epidermal thickening were significantly suppressed in Cer[NDS] d18:0/24:0-treated mice, while there was no improvement in barrier function as evaluated by TEWL (Fig. 4D–F). Immunostaining of epidermal marker proteins suggested that Cer[NDS] d18:0/24:0 normalized epidermal proliferation and hyperplasia (Fig. 4G). Topical application of Cer[NDS] d18:1/16:0 was also effective as Cer[NDS] d18:0/24:0 (supplemental Fig. S5A–D). In Spade mice at 16 weeks of age, expression levels of inflammatory genes were significantly upregulated, and topical application of Cer[NDS] to Spade mice reduced those levels, suggesting inflammatory status was significantly suppressed in Spade mice treated with Cer[NDS] (supplemental Fig. S5E).

## DISCUSSION

In this study, using global lipidome profiling, we unveiled the dysregulated sphingolipid metabolism observed during dermatitis progression caused by the constitutive activation of Jak1. Cer[NDS] with VLCFAs (C22 or more) were drastically downregulated in Spade mice before disease onset. These alterations illustrate the potentially novel pathogenic function of Jak1-dependent dermatitis pathology.

We used a mouse model of Jak1 activity-dependent dermatitis to understand the pathogenesis of dermatitis at the molecular level. JAK inhibitors have already been approved as human AD drugs, and many JAK inhibitors have advanced to clinical studies (44). Two reports have indicated that increased Jak1 activity led to an inflammatory skin phenotype in mice (45, 46). Therefore, it is suggested that Jak1 is important for maintaining skin homeostasis. In Spade mice, the homozygous R878H mutation in the kinase domain leads to the activation of Jak1 (33). This mouse model showed enhanced phosphorylation of Stat proteins in the upper epidermis. Furthermore, bone marrow chimera experiments revealed that the development of dermatitis required the expression of the Spade allele by nonhematopoietic cells. Altogether, these results suggest that excessive Jak1 activation in the epidermis is involved in the pathogenesis of AD. Further, we clarified that in the epidermis, Cer[NDS] containing VLCFAs were downregulated in the condition of Spade mice inflammation mediated by Jak1 activation. There is no previous report describing the relationship between the JAK1 pathway and ceramide metabolism. Although some reports show that the cytokine, activating broad pathways/signals including the JAK1 pathway, altered ceramide metabolism in vitro experiments in keratinocytes, JAK1-dependency is not addressed (32, 47, 48, 49). Thus, this is the first report showing dysregulated ceramide metabolism via constitutive activation of Jak1 in progressive dermatitis in mice.

The skin lipid metabolic network elaborately regulates epidermal homeostasis by each lipid molecule exerting its characteristic bioactivity or physiological function. Dysregulated lipid metabolism is observed in the skin of patients with AD. However, it remains unknown how lipid profiles are altered during the pathogenesis of AD. In this study, we demonstrated the global lipid profiling accompanying dermatitis development using untargeted and targeted lipidomic analyses. After dermatitis onset in Spade mice, various lipid metabolic changes occurred, similar to lipid changes in the human AD region, especially in ceramide, LPC, and FA (supplemental Fig. S2C, D, F, and G). In addition, these lipid species showed a proportional change in carbon chain length, that is, a decline in VLC saturated lipids (Cer[NS] 42:1;O2–50:1;O2, LPCs 24:0–26:0, and FAs 22:0–28:0) and an increase in LC lipids (Cer[NS] 32:1;O2–40:1;O2, and LPCs 16:0, 18:0). VLCFAs and ceramides are important components of the stratum corneum lipid lamellae that maintain the skin barrier. In fact, VLCFA and ceramide metabolic aberrancy cause skin barrier disruption, resulting in neonatal lethality in mice and parakeratosis in humans (9, 12, 15, 16, 49, 50, 51). Thus, in Spade mice after AD onset, aberrant VLC-lipid composition would rupture the lipid lamellar order, triggering further exacerbation of the barrier function and disease. Berdyshev *et al.* showed the gene expression pattern of ELOVL family members 1–7 in the epidermis of patients with AD, indicating that the mRNA levels of *ELOVL3/6* are downregulated in the human AD region. (32). ELOVL3 forms LC and VLC FAs (C18–C24) from their short-chain precursors, whereas ELOVL6 forms LC FAs (C12–C18) (52, 53). They showed that ELOVL3/6 contributed to the decline in VLC lipids (C24:0 in all glycerolipids, Cer[NS] 42:1;O2–44:1;O2, and SM 42:1;O2) and an increase in LC lipids (C16:0 in all glycerolipids) in keratinocytes. In the ear of Spade mice at 10 weeks of age, the level of *Elovl6* was markedly reduced, and *Elovl3/4/5* showed a decreasing trend (supplemental Fig. S2H). ELOVL4, which is essential for skin barrier function, is responsible for the formation of VLCFAs (C28 or more), whereas ELOVL5 is responsible for the formation of LC polyunsaturated FAs (52, 53). Therefore, it is possible that in Spade mice after AD onset, decreased levels of *Elovl3/4/6* are responsible for the change in the chain length of lipids (Cer[NS], LPC, and FA).

Before dermatitis onset in Spade mice at 4 weeks of age, VLCFA-containing Cer[NDS] were selectively reduced in the epidermis (Fig. 2A–C). Considering VLC-type reduction, we speculated that the enzyme activity of Elovl4 and/or CerS3 was affected. However, we assume the activity of Elovl4 is not downregulated in Spade mice at 4 weeks of age because the chain length of fatty acyls in Cer[NS] and free FAs are not changed. Spt could be also affected because DHS content was decreased in the Spade epidermis (supplemental Fig. S3A). Further, Cer[NDS] reduction could be attributed to the increased activity of Degs1, an enzyme responsible for Cer[NDS] desaturation. Thus, we measured these enzyme activities using skin homogenates and found that only Degs1 activity was upregulated (Fig. 3B–D). Δ4-dihydroceramide desaturase DEGS1 is the enzyme that converts Cer[NDS] to Cer[NS]. When DEGS1 enzyme activity is enhanced, Cer[NDS] are reduced, resulting in the downregulation of Cer[NDS]/Cer[NS]. The amount of Cer[NDS] and the ratio of Cer[NDS] to Cer[NS] were downregulated before AD onset in Spade mice, although the amount of Cer[NS] did not change significantly (Fig. 2C, D). The reduced amount of Cer[NDS] (30 nmol/mg tissue vs. WT) in Spade mice appears to be reflected in the increased amount of SM (90 nmol/mg tissue vs. WT), the endo product of Cer[NS] (supplemental Fig. S3C–E). Therefore, it is possible that Cer[NS] produced by Degs1 activation was subsequently metabolized into SM. Thus, Degs1 is a candidate for the downregulation of Cer[NDS] and the Cer[NDS]/Cer[NS] ratio in Spade mice before AD onset. Furthermore, in patients with AD, the ratio of DHS/Sph and Cer[NDS]/Cer[NS] was decreased and exhibited a strong association with disease severity (scoring AD, SCORAD), suggesting the importance of Cer[NDS]/Cer[NS] imbalance in AD pathology (54). In a mouse model of AD, Loiseau *et al.* also indicated that while DHS (d18:0) level was decreased significantly, Sph (d18:1) content was increased significantly in the stratum corneum of AD model mice, resulting in a significant downregulation of the DHS/Sph ratio (55). Chaurasia *et al.* generated liver or adipose tissue-specific *Degs1* deleted mice (56). These mice showed an increase in the Cer[NDS]/Cer[NS] ratio from 16 to 24 carbon chain length FAs in the liver, white adipose tissue, soleus, and serum. These results suggest that Degs1 recognizes Cer[NDS] with 16–24 carbon chain FAs as substrates (56). This is consistent with our results on ceramide desaturation enhancement using C16:0 and C24:0 fatty acyl-CoA (Figs. 3E and S4C). Therefore, the reduction in VLCFA-containing Cer[NDS] in Spade mice was not likely to be caused by the substrate specificity of Degs1. Hence, we hypothesized that the reduction in Cer[NDS] containing VLCFAs could be attributed to the enhancement of Degs1 activity in the differentiated keratinocytes, the site of lamellar lipid synthesis. The major component of Cer[NDS] in lamellar lipids is VLC Cer[NDS] containing C24 or more carbon chain length FAs, with very little LC Cer[NDS] containing C16 or C18 present (57). These VLC ceramides, which form lipid lamellae, are synthesized in differentiated keratinocytes in stratum spinosum or stratum granulosum. When DEGS1 activity was enhanced in the upper epidermis, VLCFA-containing Cer[NDS] levels would be selectively reduced.

Wigger *et al.* established an in vitro protein assay using rat liver microsomes to monitor de novo synthesis of sphingolipid by using deuterium-labeled chemicals (58). Using this method, we analyzed the activities of sphingolipid metabolic enzymes in tissues. We found that ceramide desaturation was enhanced approximately 2-fold higher in Spade tissue homogenates than in WT with no change in Degs1 protein expression level (Fig. 3E, G), suggesting that the enhancement of ceramide desaturation activity was attributed to its relative activity rather than protein expression level. DEGS1 is an ER membrane protein that contains three consensus motifs characteristic of membrane lipid desaturases (59, 60, 61). DEGS1 enhances its enzyme activity by myristoylation of the N-terminal domain (62). In addition, DEGS1 is phosphorylated at the 307 serine residue, although the effect of phosphorylation on enzyme activity remains unknown (63). Any posttranslational modifications may enhance the activity of Degs1 in Spade mice.

In Spade mice at 4 weeks of age, TEWL increased significantly (Fig. 1C). Further, although the normal lamellar structure was observed in Spade mice as well as WT, some aggregates, like the remaining substances of lamellar bodies, were found only in Spade lipid lamellae occasionally (Fig. 1B). These structures are observed in other skin barrier defect mice with lamellar lipid abnormalities such as *Pnpla1* or *Abhd5* knockout mice (21, 36). Therefore, it is suggested that JAK1 activation and dysregulated ceramide metabolism led to the abnormal aggregates in the lipid lamellae, which contributed to the barrier abnormality exhibited in Spade mice at 4 weeks of age.

Topical application of Cer[NDS] significantly suppressed inflammatory gene expression and epidermal thickening without affecting skin barrier function evaluated by TEWL, resulting in ameliorating dermatitis onset and progression in Spade mice (Figs. 4A–G and S5A–E). Therefore, we speculated that topically applied Cer[NDS] affected local inflammatory status and keratinocyte proliferation/differentiation without affecting skin barrier function. We found that epidermal hyperplasia in Spade mice was suppressed by Cer[NDS] d18:0/24:0 application (Fig. 4F, G). Also, the decline of VLC Cer[NDS] (d18:0/24:0, 39%; d18:0/26:0, 17% vs. WT) resulted in the reduction of total Cer[NDS] content (47% vs. WT) because VLC Cer[NDS] were the major intrinsic Cer[NDS] in the epidermis (Figs. 2B and S3C). Topical application of Cer[NDS] might ameliorate epidermal hyperplasia independent of its carbon chain length by compensating for the reduction of total intrinsic Cer[NDS] content. Further studies will be required to elucidate the underlying mechanisms behind Cer[NDS] ameliorating dermatitis.

Taken together, we revealed for the first time that dysregulated lipid metabolism, namely Degs1-associated aberrant ceramide metabolism, occurs during AD pathogenesis in Jak1-associated progressive dermatitis. Our results provide novel insights into the causal relationship between ceramide metabolism and homeostasis in the skin and propose that ceramide metabolic regulation is potentially useful for ameliorating cutaneous disorder.

## Data availability

The data generated in this study are available from the corresponding author upon request. Dr Makoto Arita, Division of Physiological Chemistry and Metabolism, Graduate School of Pharmaceutical Sciences, Keio University, Tokyo, Japan. E-mail: arita-mk@pha.keio.ac.jp

The raw mass spectrometric data has been deposited in “Metabolomics Workbench”. To view dataset’s webpage, go to www.metabolomicsworkbench.org.

Study ID is “ST002195”.

## Supplemental data

This article contains supplemental data.

## Conflict of interest

The authors declare that they have no known competing financial interests or personal relationships that could have appeared to influence the work reported in this paper.
