# Supplementary material for: Dysregulated ceramide metabolism in mouse progressive dermatitis resulting from constitutive activation of Jak1
Source: J Lipid Res. 2023 Jan 11;64(2):100329. doi: 10.1016/j.jlr.2023.100329 (PMC9932461; doi:10.1016/j.jlr.2023.100329)
Supplement: Supplemental data [file mmc1.pdf]

SUPPLEMENTAL INFORMATION:

Dysregulated ceramide metabolism in mouse progressive dermatitis via constitutive activation of Jak1

Yudai Iino[1,2], Tatsuro Naganuma[1,2], Makoto Arita[1,2,3*]

[1]Division of Physiological Chemistry and Metabolism, Graduate School of Pharmaceutical Sciences, Keio University, Tokyo, Japan

[2]Laboratory for Metabolomics, RIKEN Center for Integrative Medical Sciences, Yokohama, Japan

[3]Cellular and Molecular Epigenetics Laboratory, Graduate School of Medical Life Science, Yokohama City University, Yokohama, Japan

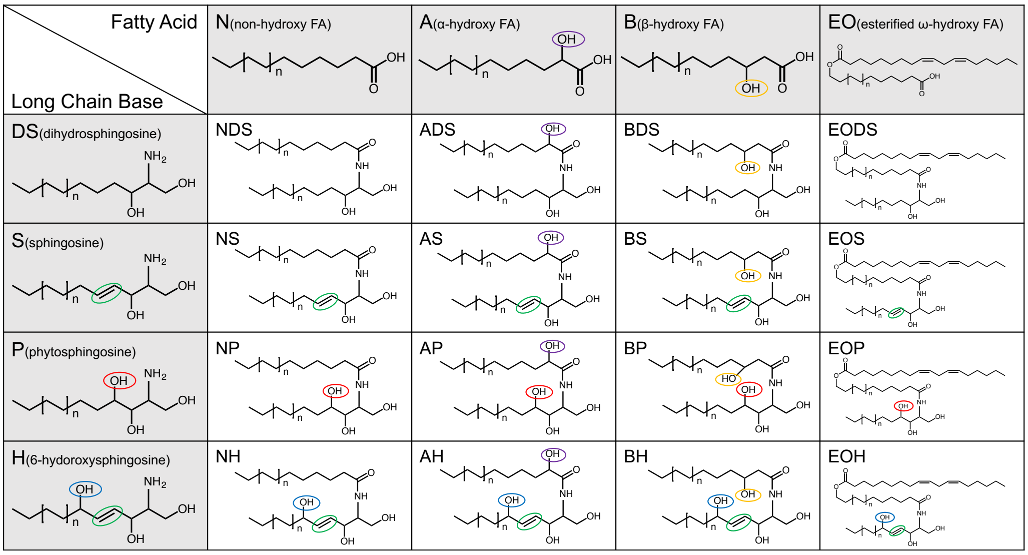

Supplemental Figure S1: Diversity of ceramide classes. Ceramides are classified as to precursor lipid class and combination.

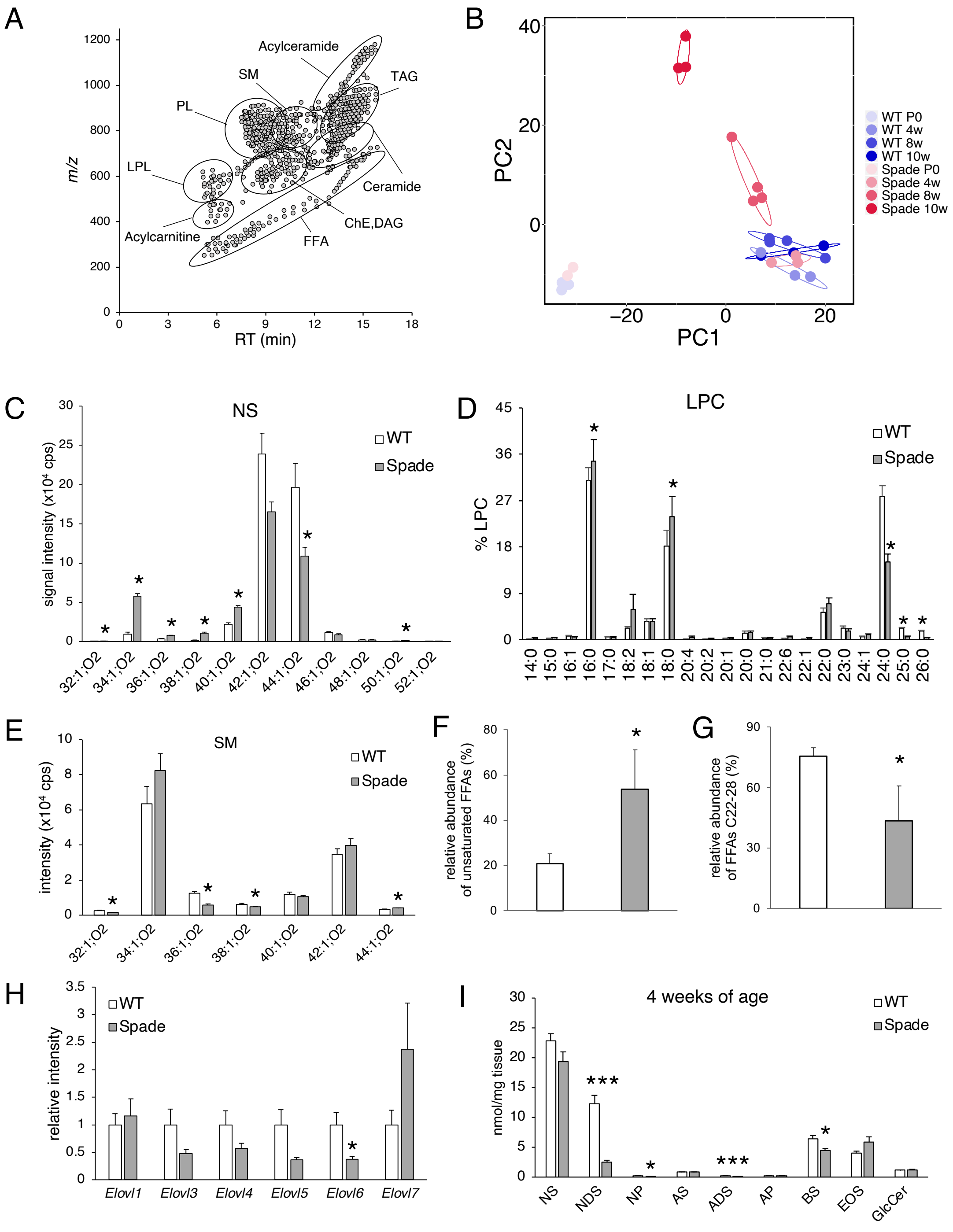
A
Acylceramide
SM
TAG
PL
LPL
Ceramide
ChE,DAG
Acylcarnitine
FFA
m/z
RT (min)
B
PC2
PC1
WT P0
WT 4w
WT 8w
WT 10w
Spade P0
Spade 4w
Spade 8w
Spade 10w
C
NS
WT
Spade
signal intensity (x10⁴ cps)
32:1;O2
34:1;O2
36:1;O2
38:1;O2
40:1;O2
42:1;O2
44:1;O2
46:1;O2
48:1;O2
50:1;O2
52:1;O2
D
LPC
% LPC
14:0
15:0
16:1
16:0
17:0
18:2
18:1
18:0
20:4
20:2
20:1
20:0
21:0
22:6
22:1
22:0
23:0
24:1
24:0
25:0
26:0
E
SM
intensity (x10⁴ cps)
F
relative abundance of unsaturated FFAs (%)
G
relative abundance of FFAs C22-28 (%)
H
relative intensity
Elovl1
Elovl3
Elovl4
Elovl5
Elovl6
Elovl7
I
4 weeks of age
nmol/mg tissue
NS
NDS
NP
AS
ADS
AP
BS
EOS
GlcCer

Supplemental Figure S2: Time-course profiling of lipid metabolic change in dermatitis development. (A) RT and *m*/*z* values of all annotated lipids in murine skin, including results for positive and negative ion modes. (B) PCA analysis was performed by use of all annotated lipid results. (C,D,E) Signal intensity of (C) Cer[NS] with saturated fatty acids, (D) LPC or (E) SM. (F) The relative level of unsaturated FFA or (G) relative abundance of FFA with chain length of C22–C28 (saturated and monounsaturated). (H) Expression levels of mRNA in ear from 10-week-old WT and Spade. Expression levels of these genes were normalized by levels of *Gapdh*. Data were expressed relative to an average of levels of each gene in WT epidermis. n=3. (I) The amounts of each ceramide class at 4 weeks of age were normalized by levels of d_9-EOS d18:1/32:0/18:2. mean + SE, student's *t*-test, *$P<0.05$, ***$P<0.001$

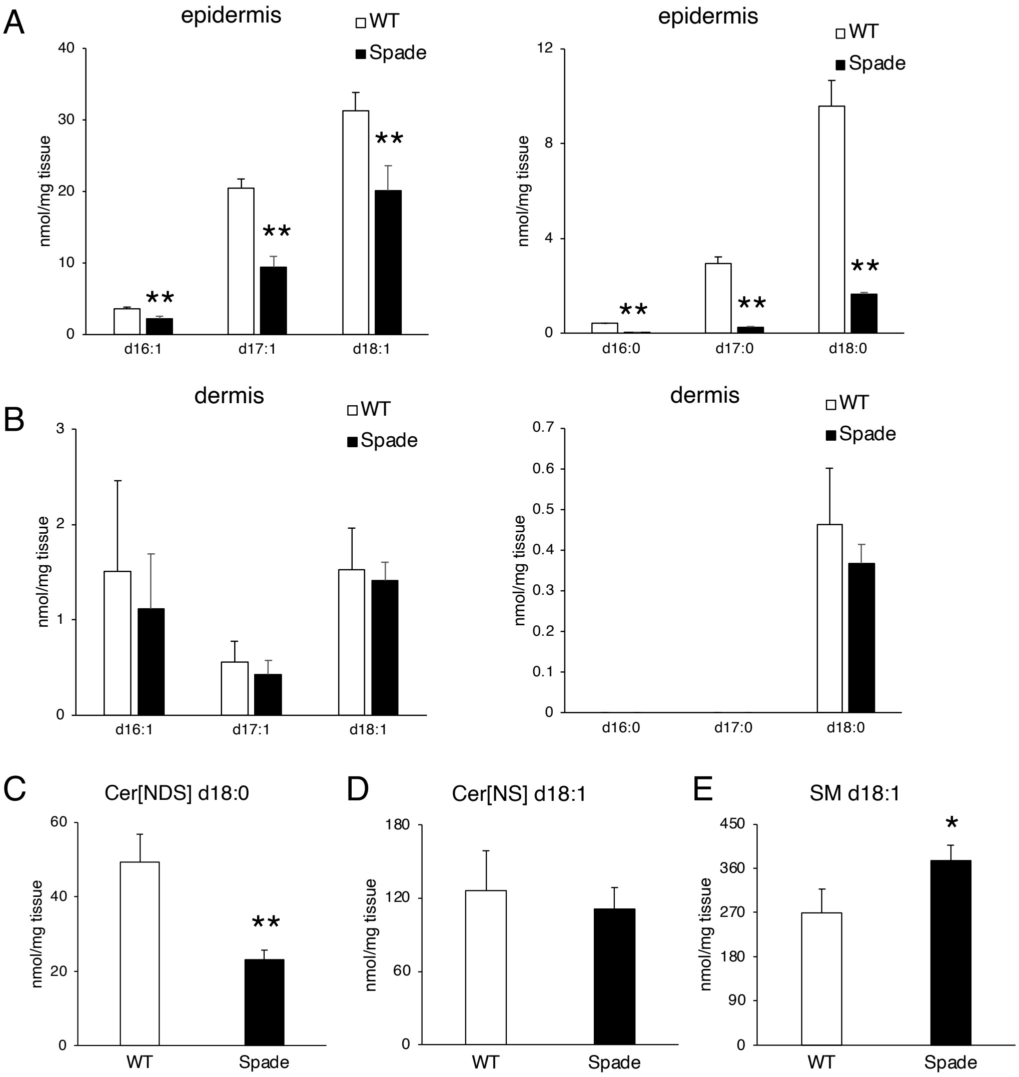

Supplemental Figure S3: Quantification of LCBs, SM, Cer[NS] and Cer[NDS] in epidermis or dermis. (A,B) Epidermis are separated from dermis from 4-week-old WT and Spade. Levels of (A) sphingosine and (B) dihydrosphingosine were expressed as concentration per ear weight. (C,D,E) The total amount of (C) Cer[NDS] d18:0, (D) Cer[NS] d18:1 and (E) SM d18:1 were expressed as concentration per ear weight. n=4., mean + SE, student's *t*-test, $*P < 0.05$, $**P < 0.01$.

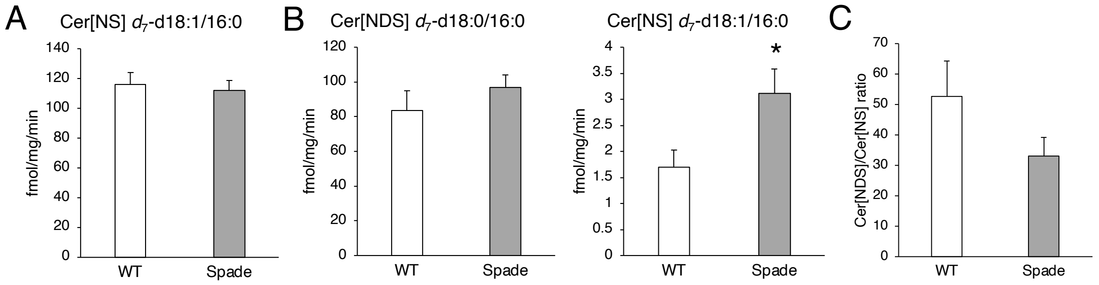

Supplemental Figure S4: Protein activity or gene examination in murine ear skin. (A,B) 20 μM C16:0-CoA was incubated for 60 min at 37˚C with 5 μM (A) d_7-Sph d18:1 or (B) d_7-DHS d18:0 in skin homogenates. NS d_7-d18:1/16:0 and NDS d_7-d18:0/16:0 were detected by LC-MS/MS. 1 mM NADH was used as a cofactor. n=3. (B) NS d_7-d18:1/16:0 was also measured as an indicator of ceramide desaturation by incubation d_7-DHS d18:0 and C16:0-CoA. (C) The product ratio of (d_7-d18:0/16:0)/(d_7-d18:1/16:0) by incubation d_7-DHS d18:0 and 16:0-CoA in ear homogenates. n=3. mean + SE, student's *t*-test, **P* < 0.05.

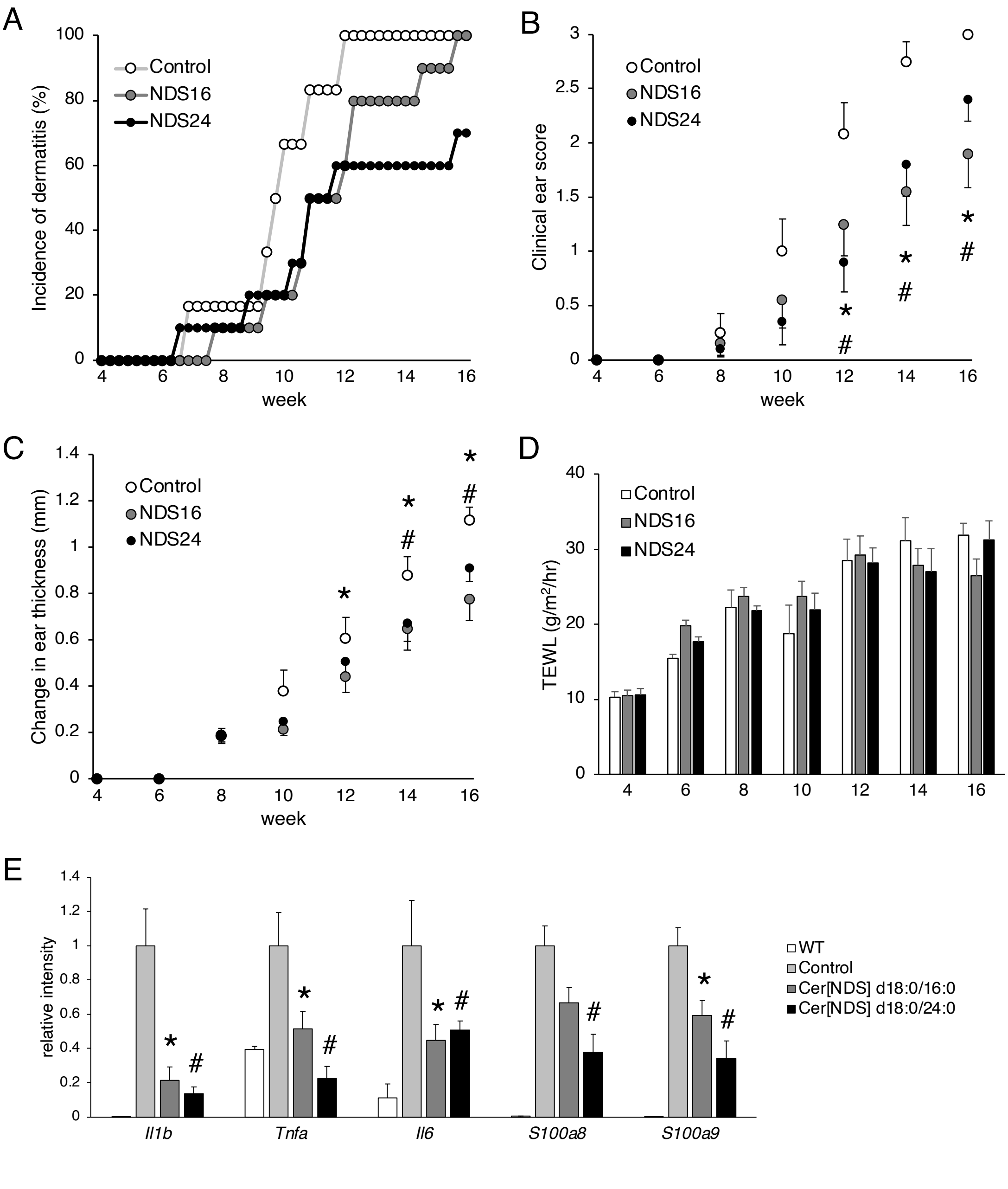

A
Incidence of dermatitis (%)
week
Control
NDS16
NDS24
B
Clinical ear score
week
Control
NDS16
NDS24
C
Change in ear thickness (mm)
week
Control
NDS16
NDS24
D
TEWL (g/m2/hr)
Control
NDS16
NDS24
E
relative intensity
Il1b
Tnfa
Il6
S100a8
S100a9
WT
Control
Cer[NDS] d18:0/16:0
Cer[NDS] d18:0/24:0

supplemental Figure S5: Analysis of Spade phenotype via the topical application of Cer[NDS] d18:0/16:0 or Cer[NDS] d18:0/24:0. (A, B) Analyses of (A) dermatitis incidence and (B) clinical scores of Spade mice treated with Cer[NDS] d18:0/16:0, Cer[NDS] d18:0/24:0 or solvent alone. Mann-Whitney's *U* test. (C, D) Analyses of (C) change in ear thickness and (D) TEWL in Spade mice treated with Cer[NDS] d18:0/16:0, Cer[NDS] d18:0/24:0 or solvent alone as a control. (E) mRNA expression levels in the ear of 16-week-old Spade mice treated with Cer[NDS] d18:0/16:0, Cer[NDS] d18:0/24:0 or solvent alone, or non-treated WT mice. Genes were analyzed via qPCR. Levels of gene expressions were normalized by levels of Gapdh. Data were expressed relative to the average levels of each gene in Spade mice treated with solvent alone (Control). Dunnett's test. n=3−20, mean ± SE, Cer[NDS] d18:0/ 24:0 vs. Control; $^{\#}P < 0.05$, Cer[NDS] d18:0/16:0 vs. Control; $^{*}P < 0.05$.

Supplemental Table S1: All MRM transitions and collision energies for ceramides and sphingomyelins.

	name	Q1	Q3	ion mode	Dwell time (msec)	Q1 Pre Bias (V)	CE	Q3 Pre Bias (V)
1	NDS d16:0/14:0	487.47	238.25	+	89	-20	-20	-21
2	NS d16:1/14:0	482.46	236.24	+	51	-20	-20	-21
3	NDS d16:0/16:0	512.5	238.25	+	51	-20	-20	-21
4	NDS d17:0/14:0	498.49	252.27	+	51	-20	-35	-21
5	NS d17:1/14:0	496.47	250.25	+	47	-20	-29	-21
6	NS d18:1/14:0	510.49	264.27	+	43	-20	-20	-21
7	NDS d16:0/18:0	540.53	238.25	+	43	-20	-27	-21
8	NS d16:1/16:0	510.49	236.24	+	43	-20	-28	-21
9	NS d17:1/16:0	524.5	250.25	+	40	-20	-25	-21
10	NDS d18:0/14:0	512.5	266.28	+	43	-20	-20	-21
11	NDS d17:0/18:0	554.55	252.27	+	43	-20	-30	-21
12	NDS d17:0/16:0	526.52	252.27	+	43	-20	-32	-21
13	NS d18:1/16:0	538.5	264.25	+	40	-20	-24	-21
14	NDS d16:0/20:0	568.57	238.25	+	40	-20	-28	-21
15	NS d16:1/18:0	538.52	236.24	+	40	-20	-25	-21
16	NDS d18:0/16:0	540.55	266.3	+	40	-20	-32	-21
17	NDS d17:0/20:0	582.58	252.27	+	40	-20	-30	-21
18	NS d17:1/18:0	552.53	250.25	+	40	-20	-25	-21
19	NDS d17:0/22:0	610.61	252.27	+	40	-20	-29	-21

20	NDS d16:0/22:0	596.6	238.25	+	40	-20	-30	-21
21	d_3-NS d18:1/18:0	569.55	264.25	+	40	-20	-23	-20
22	NS d18:1/18:0	566.55	264.25	+	40	-20	-23	-21
23	NS d16:1/20:0	566.55	236.24	+	40	-20	-24	-21
24	d_3-NDS d18:0/18:0	571.6	266.3	+	40	-22	-29	-20
25	NDS d18:0/18:0	568.55	266.3	+	40	-20	-29	-21
26	NS d17:1/20:0	580.57	250.25	+	25	-20	-26	-21
27	NS d18:1/20:0	594.6	264.25	+	40	-20	-27	-21
28	NS d17:1/22:0	608.59	250.25	+	20	-20	-27	-21
29	NS d16:1/22:0	594.58	236.24	+	24	-20	-26	-21
30	NDS d18:0/20:0	596.6	266.3	+	33	-20	-35	-21
31	NS d18:1/22:0	622.6	264.25	+	20	-20	-28	-21
32	NDS d17:0/34:1	776.78	252.27	+	24	-26	-35	-21
33	NS d16:1/24:0	622.61	236.24	+	20	-26	-26	-21
34	NDS d16:0/24:0	624.62	238.25	+	20	-20	-34	-21
35	NDS d18:0/22:0	624.65	266.3	+	20	-20	-29	-21
36	NS d17:1/24:0	636.63	250.25	+	20	-26	-29	-21
37	NDS d17:0/28:0	694.7	252.27	+	20	-26	-33	-21
38	NS d18:1/24:0	650.65	264.25	+	20	-26	-33	-21
39	NS d16:1/26:0	650.64	236.24	+	20	-26	-31	-21
40	NDS d17:0/24:0	652.64	252.27	+	20	-26	-33	-21
41	NDS d18:0/24:0	652.65	266.3	+	20	-26	-36	-21
42	NDS d16:0/26:0	652.66	238.25	+	20	-26	-36	-21

43	NDS d16:0/30:0	708.72	238.25	+	20	-26	-38	-21
44	NS d17:1/26:0	664.66	250.25	+	20	-26	-32	-21
45	NDS d17:0/26:0	666.67	252.27	+	20	-26	-36	-21
46	NS d18:1/26:0	678.65	264.25	+	20	-26	-33	-21
47	NDS d17:0/30:0	722.74	252.27	+	20	-26	-44	-21
48	NS d16:1/28:0	678.67	236.24	+	20	-26	-29	-21
49	NDS d18:0/26:0	680.7	266.3	+	20	-26	-37	-21
50	NDS d16:0/28:0	680.69	238.25	+	20	-26	-34	-21
51	NS d17:1/28:0	692.69	250.25	+	20	-26	-29	-21
52	NS d18:1/28:0	706.7	264.25	+	20	-26	-30	-21
53	NS d16:1/32:1	732.72	236.24	+	20	-26	-34	-21
54	NS d16:1/30:0	706.7	236.24	+	20	-26	-35	-21
55	NDS d18:0/28:0	708.7	266.3	+	20	-26	-40	-21
56	NS d17:1/32:1	746.74	250.25	+	20	-26	-40	-21
57	NS d17:1/30:0	720.72	250.25	+	20	-26	-31	-21
58	NS d18:1/30:0	734.75	264.25	+	21	-26	-34	-21
59	NS d16:1/32:0	734.74	236.36	+	21	-26	-31	-21
60	NDS d18:0/30:0	736.75	266.3	+	21	-26	-40	-21
61	NDS d16:0/32:0	736.75	238.25	+	21	-26	-35	-21
62	NS d17:1/32:0	748.75	250.25	+	21	-26	-35	-21
63	NS d17:1/34:1	774.77	250.25	+	21	-26	-35	-21
64	NDS d16:0/32:1	734.73	238.25	+	21	-26	-32	-21
65	NDS d17:0/32:0	750.77	252.27	+	21	-26	-48	-21

66	NS d18:1/32:0	762.75	264.25	+	21	-26	-35	-21
67	NDS d18:0/32:0	764.8	266.3	+	23	-26	-41	-21
68	NS d17:1/34:0	776.78	250.25	+	23	-26	-39	-21
69	NDS d17:0/34:0	778.8	252.27	+	27	-26	-45	-21
70	NS d17:1/36:0	804.82	250.25	+	31	-26	-39	-21
71	SM d18:1/16:0	703.5	184.1	+	22	-26	-26	-21
72	SM d18:1/18:0	731.5	184.1	+	22	-26	-28	-21
73	SM d18:1/20:0	759.6	184.1	+	22	-26	-28	-21
74	SM d18:1/22:0	787.6	184.1	+	22	-26	-30	-21
75	SM d18:1/24:0	815.6	184.1	+	22	-26	-30	-21
76	SM d18:1/26:0	843.7	184.1	+	22	-26	-34	-21
77	SM d18:1/28:0	871.7	184.1	+	22	-26	-34	-21
78	SM d18:1/30:0	899.7	184.1	+	22	-26	-35	-21
79	SM d18:1/32:0	927.8	184.1	+	22	-26	-32	-21
80	SM d18:1/34:0	955.8	184.1	+	22	-26	-35	-21
81	SM d18:1/36:0	983.8	184.1	+	22	-26	-30	-21
82	SM d18:1/30:1	897.7	184.1	+	22	-26	-30	-21
83	SM d18:1/32:1	925.7	184.1	+	22	-26	-30	-21
84	SM d18:1/34:1	953.8	184.1	+	22	-26	-30	-21

Supplemental Table S2: All MRM transitions and collision energies for LCBs.

	name	Q1	Q3	ion mode	Dwell time (msec)	Q1 Pre Bias (V)	CE	Q3 Pre Bias (V)
1	DHS d16:0	274.27	256.25	+	22	-11	-14	-22
2	$^{13}C_2$,^{15}N-DHS d16:0	277.27	259.25	+	22	-11	-14	-22
3	Sph d16:1	272.25	254.24	+	22	-11	-10	-19
4	$^{13}C_2$,^{15}N-Sph d16:1	275.25	257.24	+	22	-11	-10	-22
5	DHS d17:0	288.29	270.27	+	22	-11	-14	-22
6	d_3-DHS d17:0	291.29	273.27	+	22	-11	-14	-22
7	Sph d17:1	286.27	268.26	+	22	-11	-10	-22
8	DHS d18:0	302.3	284.3	+	22	-11	-14	-22
9	d_7-DHS d18:0	309.35	291.35	+	22	-12	-14	-16
10	$^{13}C_2$,^{15}N-DHS d18:0	305.3	287.3	+	22	-11	-14	-22
11	Sph d18:1	300.3	282.25	+	22	-11	-10	-22
12	d_7-Sph d18:1	307.35	289.35	+	22	-11	-10	-22
13	$^{13}C_2$,^{15}N-Sph d18:1	307.35	289.35	+	22	-11	-10	-22
16	d_3-NDS d18:0/18:0	571.6	266.3	+	12	-22	-30	-20
17	d_3-NS d18:1/18:0	569.55	264.25	+	12	-20	-30	-20

Supplemental Table S3: Primer list.

primer	5'→3'
h/mGAPDH_F_q	GAACGGGAAGCTCACTGGGATGGCC
h/mGAPDH_R_q	TGTCATACCAGGAAATGAGCTTGAC
mSptlc1_qPCR_F1	CCTGTCCTTTGGAGTCCTTGG
mSptlc1_qPCR_R1	GGGGAGGTAACGAAGCAGAAA
mSptlc2_qPCR_F1	GAGTATGGAGCAGGGGTGTG
mSptlc2_qPCR_R1	TGACGCATGGTTCAGCTCAT
mSptlc3_qPCR_F1	CGACGGCGACTGAAAGAGAT
mSptlc3_qPCR_R1	CCTTCTGGCAGGGAAGTAGC
mOrmdl1_qPCR_F1	TCCGGATCAGGGTAGAGCAA
mOrmdl1_qPCR_R1	ATGCAGCTGTGGCATTTTGG
mOrmdl2_qPCR_F1	GTAGGACTGCTGCATGTGGT
mOrmdl2_qPCR_R1	CCTTTCCTTGGTCAGGGGTC
mOrmdl3_qPCR_F1	AGCATTCCCTTTGTGAGCGT
mOrmdl3_qPCR_R1	CTGTGAACTGGACCCCGTAG
mKdsr_qPCR_F1	TTTGCAAACCAGAGCAGGTG
mKdsr_qPCR_R1	GCAATTGTTCGGAAAAGGCCC
mSphk1_qPCR_F1	CTCACCGAACGGAAGAACCA
mSphk1_qPCR_R1	CCTGCTCGTACCCAGCATAG
mSphk2_qPCR_F1	TCTCTGGATGAGGTCTCGGG
mSphk2_qPCR_R1	GGTGATTTCCTGGTCCCCTG
mSgpl1_qPCR_F1	AATTGTGGCTCCCGAGAGTG

mSgpl1_qPCR_R1	GTGGGGTAGAACAGACCAGC
mSgpp1_qPCR_F1	CTTGCCGCTCTACTACCTGT
mSgpp1_qPCR_R1	GGTACATGACCAGCACCCAG
mSgpp2_qPCR_F1	TGGACCCTCATCGATTCCCT
mSgpp2_qPCR_R1	TGGTCGGGCTGTAGTAGTCA
mAldh3a2_qPCR_F1	TTCTCGTAACAATAAGCTCATCAAACG
mAldh3a2_qPCR_R1	CAGCATCCCCAGCCTTCCTTTGTTG
mAldh3b2_qPCR_F1	TGAGTTCATCAACCGGCGGGAGAAGC
mAldh3b2_qPCR_R1	GTTGTTGGTTCCAGGGACCATAAGG
mElovl1_qPCR_F1	CTATCGCTTGGGCCTCGAAT
mElovl1_qPCR_R1	CTCGAACCATCCGAAGTGCT
mElovl3_qPCR_F1	GCTTTGCCATCTACACGGATGACGC
mElovl3_qPCR_R1	TCATTGGCTCTTGGATGCAACTTTGC
mElovl4_qPCR_F1	ACGTGATCATGTACTCCTACTATGG
mElovl4_qPCR_R1	CCGTTCGATGAGATACCATTCGTGG
mElovl5_qPCR_F1	AAGAACAACCACCAGATCACCGTGC
mElovl5_qPCR_R1	TCAATCCTTTCGCTGCTTCCTGGGC
mElovl6_qPCR_F1	TTCATGACTATGAACTATGGCGTGC
mElovl6_qPCR_R1	GATGTAGGCCTCAAAGAAGAAATGG
mElovl7_qPCR_F1	CTGGCTTTATTACTTCTCCAAATTC
mElovl7_qPCR_R1	GTATTTTAGTGGCGCTTGCTTTTGC
h/m-Cers3_qPCR_F1	CTGGCTTCCTCCAACAATAAAGTGG
h/m-Cers3_qPCR_R1	TCAAGTTACACTTCTTTGCCAGTCC

mDegs1_qPCR_F1	GCCGAACATTACATGTTCTTGAAGG
mDegs1_qPCR_R1	GTTCCCTTTCGGAGGCCTCCTCATC
mIl1b_qPCR_F1	TGCCACCTTTTGACAGTGATG
mIl1b_qPCR_R1	ATGTGCTGCTGCGAGATTTG
mTnfa_qPCR_F1	GATCGGTCCCCAAAGGGATG
mTnfa_qPCR_R1	CACTTGGTGGTTTGCTACGAC
mIl6_qPCR_F1	AGCCCACCAAGAACGATAGT
mIl6_qPCR_R1	TTGTGAAGTAGGGAAGGCCG
mS100a8_qPCR_F1	CCCACTTTTATCACCATCGCAA
mS100a8_qPCR_R1	AAATCACCATGCCCTCTAGAAG
mS100a9_qPCR_F1	CACCCTGAGCAAGAAGGAAT
mS100a9_qPCR_R1	TGTCATTTATGAGGGCTTCATTT

Supplemental Table S4: annotated ceramides in untargeted lipidomics (nmol/mg tissue).

Metabolites	WT P0	Spade P0	WT 4w	Spade 4w	WT 8w	Spade 8w	WT 10w	Spade 10w
Cer[NS] 32:1;O2	0.0046±0.0018	0.0058±0.00085	0.0064±0.0069	0.0087±0.0033	0.0064±0.0016	0.0077±0.0033	0.0038±0.0028	0.012±0.004
Cer[NS] 33:1;O2	0.015±0.0061	0.019±0.0014	0.03±0.0058	0.042±0.017	0.032±0.0068	0.032±0.011	0.021±0.0022	0.033±0.013
Cer[NS] 34:2;O2	0.02±0.008	0.023±0.0014	0.0095±0.0023	0.01±0.0032	0.0098±0.0018	0.014±0.0056	0.0064±0.0038	0.031±0.016
Cer[NS] 34:1;O2	0.21±0.082	0.31±0.027	0.14±0.036	0.15±0.042	0.15±0.026	0.26±0.14	0.096±0.028	0.86±0.37
Cer[NS] 35:2;O2	0.0033±0.0021	0.0064±0.00098	0.0016±0.0037	0.0068±0.0047	0.0016±0.0028	0.0023±0.0095	0.0012±0.0011	0.0021±0.0069
Cer[NS] 35:1;O2	0.01±0.0044	0.017±0.002	0.0099±0.0012	0.014±0.0054	0.011±0.0034	0.015±0.0057	0.0066±0.0041	0.018±0.0063
Cer[NS] 36:3;O2	0.0021±0.0014	0.004±0.0063	0.0017±0.0042	0.0052±0.0033	0.0011±0.002	0.0023±0.0086	0.00066±0.0017	0.0065±0.0024
Cer[NS] 36:2;O2	0.027±0.012	0.042±0.006	0.011±0.0023	0.02±0.01	0.011±0.0027	0.015±0.0052	0.0075±0.0033	0.022±0.0079
Cer[NS] 36:1;O2	0.068±0.024	0.1±0.0017	0.057±0.01	0.061±0.018	0.062±0.019	0.092±0.03	0.041±0.0028	0.12±0.041
Cer[NS] 37:2;O2	0.00049±0.0003	0.00093±0.00026	0.0027±0.0052	0.014±0.0099	0.0037±0.0064	0.0052±0.0024	0.0016±0.0026	0.0036±0.011
Cer[NS] 37:1;O2	0.0037±0.0024	0.0057±0.0013	0.0059±0.0054	0.014±0.0068	0.0064±0.0015	0.024±0.0074	0.0047±0.0037	0.029±0.0073
Cer[NS] 38:2;O2	0.0049±0.0017	0.0072±0.00044	0.007±0.0095	0.023±0.015	0.0072±0.0012	0.013±0.0049	0.0046±0.0018	0.021±0.006
Cer[NS] 38:1;O2	0.021±0.008	0.03±0.003	0.031±0.0041	0.042±0.012	0.029±0.0086	0.086±0.032	0.02±0.00087	0.15±0.041
Cer[NS] 39:1;O2	0.017±0.0071	0.024±0.0018	0.025±0.002	0.037±0.01	0.026±0.0085	0.06±0.02	0.016±0.00057	0.051±0.015
Cer[NS] 40:2;O2	0.0064±0.0025	0.011±0.0025	0.0056±0.0014	0.0078±0.0033	0.0061±0.0014	0.0083±0.0028	0.0039±0.0041	0.014±0.0057
Cer[NS] 40:1;O2	0.41±0.2	0.53±0.12	0.37±0.084	0.43±0.12	0.31±0.11	0.47±0.17	0.23±0.0079	0.63±0.2
Cer[NS] 41:1;O2	1.1±0.41	1.4±0.2	1.7±0.11	1.5±0.17	1.3±0.48	0.95±0.35	0.88±0.049	0.46±0.18
Cer[NS] 42:3;O2	0.043±0.012	0.073±0.0012	0.039±0.0066	0.034±0.0077	0.043±0.01	0.062±0.026	0.03±0.0064	0.2±0.12
Cer[NS] 42:2;O2	0.39±0.14	0.56±0.0033	0.29±0.063	0.25±0.062	0.29±0.07	0.43±0.18	0.18±0.027	0.89±0.44
Cer[NS] 42:1;O2	4.9±1.2	6±0.13	4.7±0.86	4.6±1	3.8±1.5	2.7±0.95	2.4±0.032	2.3±0.85
Cer[NS] 43:2;O2	0.038±0.016	0.049±0.0056	0.022±0.0042	0.017±0.0035	0.019±0.0048	0.019±0.007	0.013±0.00099	0.022±0.0086
Cer[NS] 43:1;O2	5.6±1.6	6.5±0.43	8.2±1.7	6.1±1.8	6.3±2.6	3.1±1.2	4.4±0.084	1.2±0.6
Cer[NS] 44:2;O2	0.16±0.048	0.18±0.0061	0.085±0.017	0.079±0.019	0.057±0.02	0.067±0.026	0.041±0.0021	0.12±0.034
Cer[NS] 44:1;O2	7.7±1.1	9±0.58	5.8±1.3	5±1.5	3.2±1.5	2.4±0.86	2±0.18	1.6±0.7
Cer[NS] 45:1;O2	0.52±0.12	0.61±0.014	0.59±0.15	0.47±0.12	0.4±0.22	0.24±0.1	0.22±0.01	0.13±0.068
Cer[NS] 46:2;O2	0.025±0.0059	0.031±0.0043	0.013±0.0014	0.0089±0.0033	0.0081±0.0039	0.0094±0.0053	0.0051±0.0072	0.025±0.0079
Cer[NS] 46:1;O2	0.54±0.11	0.57±0.012	0.37±0.1	0.31±0.092	0.19±0.11	0.16±0.064	0.1±0.0094	0.12±0.058

Cer[NS] 47:1;O2	0.087±0.026	0.11±0.011	0.06±0.0092	0.053±0.016	0.041±0.021	0.023±0.011	0.021±0.0015	0.013±0.0054
Cer[NS] 48:2;O2	0.022±0.0066	0.027±0.00095	0.007±0.00092	0.0081±0.0043	0.0055±0.0027	0.0073±0.0036	0.0044±0.00077	0.025±0.012
Cer[NS] 48:1;O2	0.14±0.02	0.15±0.0021	0.078±0.015	0.077±0.022	0.043±0.028	0.038±0.017	0.019±0.0026	0.027±0.012
Cer[NS] 49:1;O2	0.029±0.012	0.042±0.0019	0.019±0.0045	0.019±0.0059	0.013±0.0067	0.011±0.0053	0.0052±0.001	0.0048±0.0024
Cer[NS] 50:3;O2	0.0079±0.003	0.013±0.0045	0.0085±0.0035	0.0074±0.0058	0.0071±0.0031	0.0082±0.0042	0.0069±0.0024	0.018±0.0071
Cer[NS] 50:2;O2	0.03±0.0092	0.039±0.0035	0.011±0.0015	0.011±0.0037	0.0084±0.0043	0.013±0.0082	0.0046±0.0005	0.05±0.018
Cer[NS] 50:1;O2	0.039±0.0074	0.047±0.0039	0.026±0.0062	0.029±0.0079	0.016±0.01	0.024±0.0095	0.0071±0.00084	0.017±0.0067
Cer[NS] 52:3;O2	0.0041±0.00064	0.0069±0.00019	0.0043±0.0011	0.0038±0.0016	0.0033±0.0018	0.0051±0.004	0.0023±0.00053	0.019±0.0067
Cer[NS] 52:2;O2	0.028±0.0097	0.04±0.0062	0.015±0.0018	0.016±0.0041	0.013±0.0075	0.02±0.0086	0.0056±0.00036	0.037±0.013
Cer[NS] 52:1;O2	0.0039±0.0012	0.0052±0.000012	0.0069±0.0013	0.0077±0.0017	0.0064±0.003	0.008±0.0028	0.0039±0.00032	0.0052±0.0017
Cer[NS] 53:2;O2	0.0061±0.0028	0.009±0.0018	0.0047±0.00029	0.0053±0.0016	0.0081±0.0048	0.0054±0.0031	0.0028±0.001	0.0028±0.0013
Cer[NS] 54:3;O2	0.0033±0.001	0.0042±0.00012	0.0034±0.00064	0.0023±0.00052	0.002±0.0012	0.0029±0.0015	0.00091±0.00019	0.0052±0.0016
Cer[NS] 54:2;O2	0.0099±0.0034	0.013±0.0016	0.022±0.0038	0.019±0.0051	0.022±0.012	0.024±0.0092	0.011±0.00036	0.021±0.0076
Cer[NS] 56:2;O2	0.0025±0.00075	0.004±0.0017	0.012±0.0018	0.011±0.0033	0.012±0.0048	0.011±0.0039	0.0085±0.0011	0.0056±0.0014
Cer[NDS] 34:0;O2	0.0016±0.0027	N.D	0.029±0.0035	0.025±0.007	0.018±0.0059	0.015±0.0072	0.011±0.0018	0.079±0.051
Cer[NDS] 36:0;O2	0.007±0.0028	0.0068±0.00062	0.17±0.0089	0.16±0.031	0.13±0.038	0.1±0.026	0.085±0.013	0.081±0.043
Cer[NDS] 38:0;O2	0.0038±0.0015	0.005±0.0012	0.18±0.015	0.17±0.04	0.14±0.039	0.11±0.028	0.092±0.012	0.081±0.041
Cer[NDS] 40:0;O2	0.065±0.017	0.087±0.056	0.21±0.035	0.13±0.03	0.26±0.092	0.097±0.037	0.13±0.0046	0.092±0.038
Cer[NDS] 41:0;O2	0.16±0.046	0.2±0.0034	0.49±0.15	0.12±0.04	0.5±0.26	0.088±0.037	0.33±0.025	0.061±0.025
Cer[NDS] 42:0;O2	0.7±0.13	0.92±0.021	2.2±0.47	0.58±0.19	2±1.1	0.35±0.16	1.2±0.057	0.31±0.14
Cer[NDS] 43:0;O2	0.81±0.19	1.1±0.039	3.4±1.1	0.45±0.17	3.4±2	0.34±0.18	2.3±0.22	0.18±0.12
Cer[NDS] 44:0;O2	1.8±0.27	2.2±0.16	5.3±1.8	0.77±0.25	3.3±2.2	0.34±0.18	1.8±0.4	0.3±0.18
Cer[NDS] 45:0;O2	0.12±0.018	0.13±0.015	0.37±0.12	0.042±0.015	0.38±0.31	0.031±0.016	0.22±0.043	0.023±0.014
Cer[NDS] 46:0;O2	0.074±0.0035	0.082±0.0019	0.13±0.047	0.0077±0.0017	0.083±0.066	0.000058±0.00012	0.036±0.0097	N.D
Cer[NDS] 47:0;O2	0.0053±0.002	0.007±0.0037	0.051±0.015	0.00037±0.00064	0.047±0.037	0.00033±0.00066	0.02±0.0061	N.D
Cer[NDS] 48:0;O2	0.014±0.0053	0.016±0.0026	0.0095±0.0026	N.D	0.0034±0.0068	N.D	N.D	N.D
Cer[NP] 34:0;O3	N.D	N.D	0.019±0.0038	0.026±0.0062	0.034±0.012	0.022±0.01	0.028±0.0055	0.012±0.0067
Cer[NP] 35:0;O3	N.D	N.D	0.012±0.0024	0.014±0.0023	0.014±0.0051	0.014±0.0063	0.0093±0.0019	0.0057±0.0029
Cer[NP] 36:0;O3	0.0015±0.00028	0.002±0.0051	0.0043±0.0087	0.0047±0.0012	0.0046±0.0013	0.0057±0.0016	0.0035±0.00061	0.0082±0.0017

Cer[NP] 37:0;O3	N.D	N.D	0.0016±0.0004	0.0021±0.00031	0.0021±0.00073	0.004±0.0013	0.002±0.00029	0.007±0.0025
Cer[NP] 38:0;O3	N.D	N.D	0.0036±0.00098	0.0034±0.00095	0.0036±0.0014	0.0082±0.004	0.0033±0.00029	0.02±0.0052
Cer[NP] 39:0;O3	N.D	N.D	0.0055±0.00098	0.0046±0.00066	0.005±0.0024	0.0075±0.003	0.0042±0.00097	0.0095±0.0058
Cer[NP] 41:0;O3	0.025±0.0097	0.05±0.0048	0.082±0.026	0.041±0.013	0.074±0.045	0.044±0.017	0.058±0.0026	0.063±0.017
Cer[NP] 50:2;O3	0.0011±0.00046	0.0012±0.000065	0.0019±0.00059	0.0013±0.00052	0.0015±0.00097	0.0028±0.0025	0.00094±0.00033	0.014±0.0031
Cer[NP] 50:1;O3	N.D	N.D	N.D	N.D	N.D	0.00058±0.0012	N.D	0.012±0.0083
Cer[AS] 32:1;O3	0.13±0.04	0.16±0.044	0.1±0.015	0.11±0.019	0.084±0.02	0.063±0.022	0.065±0.0093	0.03±0.011
Cer[AS] 33:1;O3	0.15±0.045	0.18±0.004	0.34±0.046	0.31±0.069	0.31±0.088	0.31±0.11	0.23±0.035	0.14±0.056
Cer[AS] 34:2;O3	0.0077±0.0017	0.009±0.0013	0.006±0.0046	0.0067±0.0012	0.0056±0.0015	0.014±0.0047	0.0047±0.002	0.014±0.005
Cer[AS] 34:1;O3	0.37±0.12	0.41±0.0089	0.32±0.053	0.31±0.11	0.2±0.061	0.47±0.17	0.16±0.022	0.57±0.16
Cer[AS] 35:1;O3	0.018±0.0056	0.026±0.0032	0.019±0.0022	0.025±0.0047	0.018±0.0038	0.033±0.011	0.012±0.00039	0.032±0.016
Cer[AS] 36:1;O3	0.011±0.0036	0.015±0.0058	0.014±0.0013	0.019±0.0051	0.013±0.0035	0.044±0.014	0.01±0.0011	0.042±0.0097
Cer[AS] 38:1;O3	0.00025±0.00012	0.00032±0.00016	0.0012±0.00023	0.0015±0.00012	0.0014±0.00029	0.012±0.0046	0.001±0.00022	0.021±0.0034
Cer[AS] 38:1;O3	N.D	N.D	0.0035±0.00051	0.0063±0.0017	0.0035±0.00067	0.015±0.0054	0.0033±0.00043	0.026±0.0064
Cer[AS] 40:1;O3	0.0015±0.00059	0.002±0.00046	0.0021±0.00036	0.0027±0.0003	0.0023±0.00061	0.0096±0.0032	0.0019±0.00028	0.016±0.0039
Cer[AS] 42:1;O3	0.11±0.037	0.13±0.048	0.16±0.03	0.16±0.014	0.18±0.045	0.18±0.062	0.14±0.01	0.31±0.15
Cer[AS] 49:1;O3	0.054±0.016	0.068±0.013	0.034±0.0098	0.04±0.012	0.037±0.015	0.036±0.014	0.029±0.0017	0.012±0.0067
Cer[AS] 50:1;O3	0.21±0.041	0.25±0.011	0.076±0.019	0.089±0.024	0.07±0.031	0.083±0.031	0.047±0.0012	0.044±0.019
Cer[AS] 51:2;O3	0.049±0.017	0.064±0.011	0.025±0.0059	0.024±0.0055	0.026±0.0082	0.027±0.009	0.017±0.0013	0.016±0.005
Cer[AS] 51:1;O3	0.033±0.011	0.047±0.0071	0.05±0.016	0.048±0.013	0.059±0.024	0.056±0.022	0.046±0.0027	0.027±0.014
Cer[AS] 52:2;O3	0.19±0.067	0.24±0.014	0.059±0.015	0.065±0.015	0.05±0.017	0.092±0.031	0.037±0.0029	0.14±0.043
Cer[AS] 52:1;O3	0.04±0.011	0.063±0.014	0.036±0.0054	0.045±0.012	0.031±0.013	0.049±0.02	0.024±0.0035	0.023±0.011
Cer[AS] 53:2;O3	0.029±0.0075	0.041±0.0028	0.027±0.0034	0.027±0.0044	0.033±0.011	0.032±0.011	0.025±0.002	0.016±0.0066
Cer[AS] 54:2;O3	0.051±0.022	0.068±0.0071	0.036±0.0067	0.037±0.0098	0.034±0.013	0.045±0.016	0.025±0.0016	0.035±0.014
Cer[ADS] 32:0;O3	0.0026±0.00086	0.0039±0.0002	0.0094±0.0016	0.0051±0.0012	0.011±0.0041	0.0034±0.0014	0.0081±0.0018	0.0016±0.00076
Cer[ADS] 33:0;O3	0.026±0.0098	0.037±0.0022	0.061±0.012	0.025±0.0051	0.067±0.025	0.026±0.0092	0.051±0.01	0.015±0.0056
Cer[ADS] 34:0;O3	0.058±0.021	0.079±0.0055	0.081±0.018	0.027±0.0073	0.058±0.023	0.032±0.013	0.04±0.009	0.027±0.011
Cer[ADS] 36:0;O3	0.0055±0.0021	0.0069±0.00074	0.012±0.0033	0.0095±0.0034	0.0088±0.0022	0.012±0.0042	0.0061±0.0012	0.013±0.0052
Cer[ADS] 41:1;O3	0.014±0.0057	0.021±0.0046	0.024±0.0024	0.028±0.0046	0.027±0.009	0.06±0.021	0.022±0.0016	0.051±0.015

Cer[ADS] 42:1;O3	0.019±0.0073	0.024±0.0034	0.019±0.0027	0.023±0.0044	0.018±0.0063	0.029±0.0096	0.015±0.0011	0.033±0.011
Cer[ADS] 42:0;O3	0.13±0.04	0.17±0.031	0.37±0.088	0.15±0.036	0.38±0.2	0.17±0.067	0.27±0.0082	0.53±0.17
Cer[ADS] 43:1;O3	0.37±0.16	0.48±0.091	0.52±0.092	0.48±0.11	0.54±0.22	0.52±0.2	0.41±0.016	0.22±0.089
Cer[ADS] 43:0;O3	0.17±0.036	0.24±0.045	0.54±0.18	0.11±0.025	0.62±0.33	0.11±0.054	0.48±0.034	0.13±0.051
Cer[ADS] 44:1;O3	0.95±0.23	1.1±0.06	0.55±0.14	0.54±0.14	0.33±0.11	0.61±0.19	0.26±0.013	0.55±0.17
Cer[ADS] 44:0;O3	0.32±0.099	0.43±0.11	0.54±0.18	0.16±0.045	0.42±0.27	0.16±0.088	0.29±0.018	0.26±0.084
Cer[AP] 33:0;O4	0.0014±0.00047	0.0022±0.00018	0.0077±0.0085	0.011±0.0034	0.01±0.0041	0.0086±0.0031	0.0079±0.0016	0.0074±0.0061
Cer[AP] 34:0;O4	N.D	N.D	0.023±0.0049	0.04±0.012	0.033±0.014	0.033±0.014	0.025±0.004	0.014±0.007
Cer[AP] 35:0;O4	N.D	N.D	0.0098±0.0013	0.018±0.0025	0.012±0.0043	0.011±0.004	0.0079±0.0017	0.003±0.0014
Cer[AP] 36:0;O4	N.D	N.D	0.0044±0.006	0.0051±0.0081	0.0047±0.0016	0.0052±0.0018	0.0033±0.00088	0.0023±0.0011
Cer[AP] 38:0;O4	N.D	N.D	0.0024±0.0061	0.003±0.00082	0.0024±0.00085	0.0031±0.0014	0.0017±0.00024	0.0091±0.0033
Cer[AP] 40:0;O4	0.0021±0.0012	0.0034±0.00039	0.011±0.0032	0.012±0.0023	0.0087±0.0027	0.018±0.0081	0.0071±0.00099	0.022±0.011
Cer[AP] 42:1;O4	0.0046±0.0022	0.0071±0.00062	0.018±0.0012	0.025±0.0043	0.021±0.0069	0.049±0.016	0.019±0.0019	0.043±0.012
Cer[AP] 42:0;O4	0.0069±0.0024	0.0092±0.00034	0.013±0.00069	0.0079±0.0032	0.014±0.0064	0.014±0.0073	0.009±0.0011	0.083±0.032
Cer[AP] 44:1;O4	0.015±0.0053	0.02±0.0062	0.021±0.0032	0.029±0.0034	0.021±0.0069	0.074±0.026	0.018±0.0035	0.099±0.031
Cer[AP] 44:0;O4	0.024±0.0048	0.029±0.0043	0.037±0.0094	0.027±0.0053	0.034±0.015	0.028±0.014	0.024±0.0014	0.054±0.019
Cer[BS] 33:1;O3	0.003±0.0095	0.0041±0.00014	0.013±0.0019	0.011±0.0027	0.01±0.0027	0.01±0.0035	0.0074±0.0014	0.0079±0.0029
Cer[BS] 34:1;O3	0.0079±0.0025	0.0096±0.0028	0.014±0.0038	0.016±0.0037	0.011±0.0046	0.015±0.0051	0.0077±0.0018	0.022±0.006
Cer[BS] 36:1;O3	0.002±0.0047	0.003±0.0027	0.0031±0.0067	0.0039±0.0052	0.0031±0.00072	0.01±0.0031	0.0032±0.0013	0.009±0.002
Cer[BS] 37:1;O3	N.D	N.D	0.0012±0.000072	0.0022±0.0032	0.0015±0.00041	0.012±0.0031	0.0011±0.00035	0.0095±0.0017
Cer[BS] 38:1;O3	0.0012±0.00046	0.0019±0.00015	0.0037±0.0068	0.005±0.0012	0.0044±0.0014	0.021±0.0062	0.0032±0.001	0.023±0.0061
Cer[BS] 40:1;O3	0.019±0.0072	0.024±0.0021	0.044±0.0035	0.045±0.008	0.049±0.02	0.056±0.018	0.038±0.0032	0.042±0.013
Cer[BS] 41:1;O3	0.057±0.02	0.074±0.0014	0.13±0.017	0.1±0.02	0.15±0.058	0.1±0.036	0.11±0.0045	0.064±0.026
Cer[BS] 41:1;O3	0.11±0.033	0.14±0.013	0.17±0.027	0.16±0.026	0.17±0.057	0.15±0.051	0.12±0.0077	0.14±0.052
Cer[BS] 42:1;O3	0.59±0.21	0.63±0.045	1.1±0.22	0.83±0.26	1.1±0.45	0.5±0.17	0.78±0.043	0.2±0.1
Cer[BS] 43:1;O3	1.5±0.33	1.6±0.28	3.1±0.67	1.9±0.46	3±1.2	1.2±0.47	2.1±0.05	0.35±0.22
Cer[BS] 44:2;O3	0.035±0.0094	0.035±0.0092	0.03±0.0082	0.023±0.0061	0.02±0.012	0.011±0.0048	0.013±0.001	0.0052±0.0029
Cer[BS] 44:1;O3	1.6±0.29	1.7±0.0018	1.6±0.38	1.1±0.27	1.1±0.58	0.51±0.17	0.66±0.11	0.21±0.13
Cer[BS] 45:1;O3	0.15±0.024	0.18±0.031	0.063±0.015	0.095±0.022	0.04±0.01	0.024±0.01	0.017±0.0038	0.019±0.0087

Cer[BS] 45:1;O3	0.078±0.03	0.09±0.0024	0.13±0.026	0.091±0.026	0.12±0.046	0.065±0.028	0.085±0.00066	0.023±0.014
Cer[BS] 46:1;O3	0.057±0.018	0.052±0.014	0.07±0.017	0.046±0.0081	0.048±0.034	0.015±0.0095	0.032±0.0017	N.D
Cer[BS] 47:1;O3	0.0019±0.00061	0.0023±0.0014	0.023±0.0055	0.0077±0.0033	0.019±0.014	0.0029±0.0028	0.0086±0.00099	N.D
Cer[EOS] 52:1;O2	N.D	N.D	N.D	N.D	N.D	0.0074±0.0062	0.00024±0.00042	0.025±0.0054
Cer[EOS] 61:3;O2	0.0023±0.000075	0.0026±0.00044	0.019±0.0044	0.0097±0.0019	0.024±0.017	0.0058±0.0029	0.013±0.0017	0.0023±0.0018
Cer[EOS] 62:3;O2	0.0092±0.0012	0.0087±0.0021	0.0075±0.0016	0.0072±0.0015	0.008±0.0063	0.003±0.0017	0.0037±0.00064	0.0017±0.0014
Cer[EOS] 62:1;O2	0.0061±0.0011	0.007±0.0046	0.0029±0.008	0.015±0.0028	0.003±0.00098	0.032±0.0086	0.0032±0.00095	0.034±0.0012
Cer[EOS] 63:3;O2	0.0034±0.00063	0.0043±0.00099	0.0044±0.0014	0.0068±0.0016	0.0052±0.0049	0.0029±0.0016	0.002±0.00048	0.00074±0.00086
Cer[EOS] 64:3;O2	0.025±0.0044	0.027±0.005	0.01±0.003	0.016±0.0038	0.0093±0.0068	0.0077±0.0038	0.0041±0.00095	0.0036±0.0022
Cer[EOS] 64:1;O2	0.003±0.0093	0.0045±0.0012	0.0058±0.0019	0.023±0.0015	0.0071±0.001	0.034±0.0075	0.012±0.0058	0.027±0.0034
Cer[EOS] 65:3;O2	0.071±0.0025	0.076±0.0043	0.067±0.017	0.12±0.034	0.071±0.051	0.062±0.031	0.033±0.0055	0.018±0.012
Cer[EOS] 66:3;O2	0.31±0.064	0.3±0.023	0.13±0.017	0.22±0.068	0.11±0.064	0.12±0.057	0.056±0.0085	0.05±0.025
Cer[EOS] 67:4;O2	0.074±0.021	0.12±0.011	0.015±0.0028	0.025±0.0075	0.013±0.0062	0.017±0.0065	0.0065±0.00064	0.014±0.0045
Cer[EOS] 67:3;O2	0.35±0.037	0.36±0.011	0.67±0.14	0.95±0.35	0.69±0.28	0.67±0.3	0.47±0.0092	0.19±0.079
Cer[EOS] 68:4;O2	0.41±0.14	0.52±0.027	0.035±0.0063	0.066±0.022	0.028±0.014	0.054±0.022	0.013±0.0031	0.097±0.044
Cer[EOS] 68:3;O2	0.78±0.14	0.8±0.029	0.7±0.18	1.1±0.41	0.52±0.2	0.77±0.28	0.36±0.027	0.36±0.093
Cer[EOS] 69:5;O2	0.073±0.026	0.1±0.021	0.017±0.0033	0.017±0.0049	0.012±0.0054	0.013±0.0062	0.0059±0.0005	0.012±0.0029
Cer[EOS] 69:4;O2	0.51±0.14	0.77±0.15	0.38±0.058	0.6±0.21	0.36±0.14	0.45±0.2	0.23±0.018	0.19±0.049
Cer[EOS] 69:3;O2	0.1±0.018	0.13±0.004	0.31±0.072	0.41±0.16	0.32±0.084	0.32±0.13	0.29±0.039	0.094±0.02
Cer[EOS] 70:5;O2	0.31±0.087	0.35±0.016	0.032±0.0062	0.04±0.011	0.014±0.0064	0.039±0.02	0.0076±0.0017	0.078±0.018
Cer[EOS] 70:4;O2	1.5±0.39	1.7±0.15	0.67±0.13	1.1±0.42	0.5±0.19	1±0.35	0.32±0.04	0.9±0.14
Cer[EOS] 70:3;O2	0.13±0.032	0.15±0.0099	0.23±0.058	0.32±0.11	0.17±0.049	0.24±0.068	0.15±0.022	0.11±0.013
Cer[EOS] 71:5;O2	0.035±0.0092	0.043±0.0037	0.037±0.0084	0.029±0.011	0.027±0.011	0.023±0.0093	0.017±0.0021	0.0075±0.0023
Cer[EOS] 71:4;O2	0.14±0.036	0.18±0.0084	0.28±0.054	0.36±0.14	0.31±0.094	0.33±0.13	0.23±0.02	0.1±0.022
Cer[EOS] 71:3;O2	0.007±0.0022	0.0076±0.00027	0.027±0.0058	0.032±0.01	0.028±0.0067	0.024±0.0087	0.027±0.0031	0.0076±0.0099
Cer[EOS] 72:5;O2	0.076±0.022	0.088±0.0044	0.048±0.013	0.046±0.017	0.029±0.012	0.046±0.017	0.018±0.0017	0.045±0.0047
Cer[EOS] 72:4;O2	0.2±0.058	0.23±0.021	0.27±0.056	0.34±0.14	0.23±0.069	0.31±0.1	0.17±0.016	0.17±0.013
Cer[EOS] 72:3;O2	0.0096±0.0031	0.013±0.0019	0.017±0.0052	0.022±0.0059	0.013±0.0034	0.014±0.0059	0.014±0.0018	0.0055±0.002
Cer[EOS] 73:5;O2	0.0047±0.0023	0.0058±0.0014	0.0094±0.0026	0.0074±0.0033	0.0075±0.0029	0.0051±0.0012	0.0053±0.00049	0.0015±0.00035

Cer[EOS] 73:4;O2	0.013±0.003	0.018±0.0023	0.039±0.0067	0.043±0.014	0.044±0.0099	0.037±0.015	0.039±0.0035	0.01±0.0034
Cer[EOS] 74:4;O2	0.018±0.005	0.022±0.002	0.031±0.0073	0.038±0.014	0.028±0.0074	0.027±0.0088	0.025±0.0019	0.012±0.00084
HexCer[NS] 34:1;O2	0.051±0.016	0.08±0.018	0.032±0.0037	0.043±0.012	0.021±0.0052	0.036±0.023	0.015±0.0038	0.079±0.027
HexCer[NS] 38:1;O2	0.011±0.0036	0.016±0.0031	0.029±0.0021	0.031±0.0074	0.026±0.0071	0.026±0.0088	0.018±0.0044	0.024±0.0065
HexCer[NS] 40:1;O2	0.059±0.019	0.078±0.0096	0.075±0.006	0.079±0.021	0.064±0.019	0.078±0.03	0.047±0.0083	0.089±0.029
HexCer[NS] 41:1;O2	0.027±0.011	0.039±0.0064	0.041±0.0046	0.042±0.012	0.039±0.013	0.041±0.015	0.028±0.0044	0.052±0.019
HexCer[NS] 42:3;O2	0.0093±0.0032	0.014±0.0015	0.027±0.0043	0.027±0.0061	0.026±0.0074	0.033±0.013	0.021±0.0044	0.039±0.016
HexCer[NS] 42:2;O2	0.059±0.016	0.084±0.014	0.11±0.0095	0.1±0.022	0.1±0.033	0.14±0.055	0.078±0.014	0.16±0.054
HexCer[NS] 42:1;O2	0.41±0.13	0.56±0.033	0.45±0.027	0.48±0.13	0.4±0.15	0.5±0.21	0.28±0.046	0.59±0.22
HexCer[NS] 43:1;O2	0.25±0.07	0.36±0.055	0.21±0.03	0.19±0.056	0.15±0.048	0.11±0.038	0.093±0.01	0.077±0.035
HexCer[NS] 44:2;O2	0.018±0.0058	0.025±0.0045	0.0087±0.00079	0.0072±0.0015	0.0071±0.0024	0.014±0.01	0.0072±0.0025	0.026±0.0089
HexCer[NS] 44:1;O2	0.31±0.077	0.4±0.0085	0.13±0.025	0.14±0.039	0.074±0.026	0.099±0.043	0.051±0.0012	0.12±0.048
HexCer[NS] 45:1;O2	0.018±0.0057	0.028±0.0045	0.0086±0.001	0.01±0.0028	0.0065±0.0015	0.0082±0.005	0.0045±0.00092	0.0056±0.0038
HexCer[NS] 46:1;O2	0.019±0.01	0.022±0.0083	0.0083±0.0023	0.0085±0.0022	0.0055±0.0025	0.0046±0.0025	0.0027±0.00097	0.0061±0.0028
HexCer[NDS] 44:0;2O	0.015±0.0014	0.014±0.0034	0.0054±0.0015	0.0024±0.00055	0.0039±0.0018	0.0018±0.00066	0.0025±0.00052	0.0026±0.0011
HexCer[NDS] 46:0;2O	0.00077±0.000053	0.00087±0.000081	0.00045±0.00011	0.00032±0.000068	0.00035±0.00022	0.00021±0.000076	0.00021±0.00035	0.00021±0.000099
HexCer[EOS] 67:5;4O	0.00047±0.00014	0.00051±0.000067	0.00014±0.000022	0.00027±0.000043	0.00018±0.0013	0.00024±0.00012	0.000088±0.000031	0.000083±0.000044
HexCer[EOS] 68:5;4O	0.00012±0.000049	0.00022±0.00005	0.00001±0.0000024	0.000017±0.0000036	0.0000069±0.0000024	0.000015±0.0000088	0.0000017±0.00000053	0.000035±0.000016
HexCer[EOS] 69:5;4O	0.00019±0.000087	0.00035±0.000013	0.0001±0.000084	0.00015±0.000029	0.0001±0.000044	0.00009±0.00004	0.000041±0.000013	0.000058±0.000027
HexCer[EOS] 70:5;4O	0.00034±0.00013	0.00058±0.000037	0.00023±0.000013	0.0003±0.000058	0.00017±0.000099	0.00028±0.00014	0.000079±0.000028	0.00032±0.00014
